# Myval Beyond the Aortic Valve: Device–Anatomy Interaction, Procedural Strategy, and Clinical Evidence in Mitral, Tricuspid, and Pulmonary Positions

**DOI:** 10.3390/bioengineering13090957

**Published:** 2026-08-22

**Authors:** Georgios E. Papadopoulos, Ilias Ninios, Sotirios Evangelou, Apostolia Marvaki, Maria Kalaitzoglou, Andreas Ioannides, Grigorios Giamouzis, Vlasis Ninios

**Affiliations:** 1 2nd Cardiology Department, Interbalkan Medical Center, 57001 Thessaloniki, Greece; iliasninios@gmail.com (I.N.); evangelousotirios@yahoo.com (S.E.); apostoliamarvaki@yahoo.com (A.M.); mariakalaitzo25@gmail.com (M.K.); ioannidesandreas69@gmail.com (A.I.); vninios@gmail.com (V.N.); 2 School of Medicine, University of Thessaly, 41110 Larissa, Greece; grgiamouzis@gmail.com

**Keywords:** Myval, balloon-expandable transcatheter heart valve, mitral valve-in-valve, mitral valve-in-ring, valve-in-mitral annular calcification, tricuspid valve-in-valve, transcatheter pulmonary valve implantation, right ventricular outflow tract

## Abstract

The Myval balloon-expandable transcatheter heart valve was developed for transcatheter aortic valve implantation, but its broad 20–32 mm size matrix and controlled deployment have prompted use in non-aortic landing zones. This narrative review critically synthesizes Myval-specific case reports, case series, and observational cohorts, together with relevant platform-level evidence, addressing device design, anatomical selection, imaging, procedural strategy, clinical outcomes, and evidence gaps. Reported applications include mitral valve-in-valve, valve-in-ring, and valve-in-mitral annular calcification; tricuspid valve-in-valve and valve-in-ring; and pulmonary implantation in conduits, surgical bioprostheses, and selected native or patched right ventricular outflow tracts. Outcomes appear most predictable within circular stented surgical bioprostheses, whereas non-circular rings, severe mitral annular calcification, and compliant or aneurysmal outflow tracts present greater risks of inadequate anchoring, paravalvular regurgitation, embolization, frame deformation, left ventricular outflow tract obstruction, and coronary compression. Intermediate and extra-large diameters increase the available nominal sizing options; however, no clinical evidence demonstrates that this reduces embolization, paravalvular regurgitation, residual gradients, or reintervention. The available data document procedural feasibility in anatomically selected patients but are predominantly observational, with limited independent adjudication and follow-up. These procedures are generally off-label and should remain individualized Heart Team decisions; prospective multicenter studies are required to define comparative safety, antithrombotic management, durability, and lifetime reintervention strategies.

## 1. Introduction

The increasing use of biological heart valve prostheses has generated a growing population of patients presenting with structural valve deterioration, non-structural dysfunction, thrombosis, or endocarditis years after the initial operation. This clinical burden extends beyond the aortic position and includes failed mitral and tricuspid bioprostheses, dysfunctional annuloplasty rings, pulmonary bioprostheses, and surgically reconstructed right ventricular outflow tracts (RVOTs) in patients with congenital heart disease [1,2]. Although repeat surgery remains the reference treatment for many patients with prosthetic valve dysfunction, reoperation may be associated with substantial procedural complexity because of advanced age, multimorbidity, previous sternotomy, adhesions, ventricular dysfunction, pulmonary hypertension, or prior complex congenital cardiac surgery [1,2,3]. Consequently, less invasive transcatheter strategies have become increasingly relevant for patients in whom the anticipated risk of repeat surgery is high or prohibitive.

The implantation of balloon-expandable transcatheter heart valves (THVs) in non-aortic positions has evolved from isolated compassionate-use procedures to a clinically relevant therapeutic option in selected anatomical settings. Transcatheter mitral valve replacement using balloon-expandable THVs can be performed within a failed surgical bioprosthesis as mitral valve-in-valve (MViV), within a failed annuloplasty ring as mitral valve-in-ring (MViR), or in selected patients with severe mitral annular calcification as valve-in-MAC (ViMAC) [4,5]. These procedures are not equivalent: MViV generally provides the most predictable anchoring and sealing, whereas MViR and ViMAC are characterized by greater anatomical heterogeneity and higher risks of paravalvular regurgitation, device migration, incomplete frame expansion, and left ventricular outflow tract obstruction [4,5]. Balloon-expandable THVs have similarly been implanted within failed tricuspid bioprostheses and rings, while transcatheter pulmonary valve implantation has become an established alternative to repeat surgery for appropriately selected patients with dysfunctional RV–pulmonary artery conduits, pulmonary bioprostheses, or selected native and patched RVOT anatomies [1,6,7].

Non-aortic implantation changes the anchoring substrate, loading conditions, and interaction with adjacent structures. A stented surgical bioprosthesis usually provides a relatively rigid, circular and reproducible landing zone, whereas an annuloplasty ring may be incomplete, non-circular, flexible, or variably resistant to deformation. Severe MAC creates an irregular and frequently discontinuous calcific landing zone adjacent to the left ventricular outflow tract, while native or patch-enlarged RVOTs may be large, compliant, aneurysmal, and dynamically variable throughout the cardiac cycle [4,5,8]. Furthermore, mitral, tricuspid, and pulmonary implants are exposed to markedly different transvalvular pressures, flow patterns, cyclic loading conditions, and relationships with adjacent structures. These differences influence valve sizing, anchoring, sealing, frame geometry, leaflet kinematics, thrombogenicity, and long-term durability. Clinical performance in a non-aortic position therefore depends on the interaction between the device and the specific landing-zone substrate rather than on the characteristics of the THV in isolation.

The Myval THV series (Meril Life Sciences, Vapi, India) is a balloon-expandable platform originally developed for transcatheter aortic valve implantation. The first-in-human Myval-1 study established the initial feasibility and hemodynamic performance of the system in native aortic stenosis [9]. Subsequently, the LANDMARK and COMPARE-TAVI 1 randomized trials demonstrated non-inferiority of the Myval THV series to contemporary transcatheter aortic platforms for their respective composite endpoints in patients undergoing transcatheter aortic valve implantation [10,11]. The platform incorporates bovine pericardial leaflets mounted on a balloon-expandable metallic frame and an external polyethylene terephthalate sealing cuff. A distinguishing feature is its broad size matrix, including intermediate diameters between conventional valve sizes and extra-large 30.5- and 32 mm prostheses [9,12]. These characteristics may permit closer dimensional matching in selected non-aortic landing zones and may expand the range of anatomies amenable to balloon-expandable valve implantation. Nevertheless, these potential advantages remain anatomically and procedure dependent, and evidence derived from aortic implantation cannot be directly extrapolated to mitral, tricuspid, or pulmonary applications.

The largest body of non-aortic Myval experience has accumulated in the mitral and pulmonary positions. Initial mixed-position valve-in-valve experience was followed by dedicated reports of transseptal MViV implantation, multicenter evaluation of left-sided valve-in-valve and valve-in-ring procedures, and a single-center series reporting one-year outcomes after Myval MViV implantation [13,14,15,16]. These studies have demonstrated procedural feasibility and generally favorable early hemodynamic performance; however, they remain retrospective, include relatively small populations, and provide limited information regarding long-term valve durability, thrombosis, and reintervention. More recent reports have extended the use of Myval to selected patients with severe MAC and have described alternative delivery strategies intended to improve support and coaxiality in technically challenging mitral anatomies [17,18]. These emerging experiences remain case based and should therefore be interpreted as demonstrations of technical feasibility rather than evidence of established clinical effectiveness.

Experience in the tricuspid position is substantially more limited. Published Myval procedures consist predominantly of individual tricuspid valve-in-valve cases, an isolated valve-in-ring report, and a small case series incorporating one-year follow-up [19,20,21,22]. Although these reports demonstrate that implantation is technically possible through transfemoral or transjugular venous access, the available evidence is insufficient to define optimal sizing, implantation depth, antithrombotic therapy, or long-term interaction with pre-existing transvenous pacing leads. In particular, Myval implantation within a failed tricuspid prosthesis or ring should be distinguished from orthotopic replacement of the native tricuspid valve using a dedicated transcatheter replacement system.

The pulmonary position is supported by several small observational experiences encompassing dysfunctional prestented conduits, surgical pulmonary bioprostheses, and native or patched RVOTs [23,24,25]. The availability of extra-large valve sizes is particularly relevant in this setting because a substantial proportion of patients after tetralogy of Fallot repair or transannular patch reconstruction have RVOT dimensions exceeding the range of conventional balloon-expandable pulmonary valves. More recent studies have reported direct implantation of 30.5 and 32 mm Myval prostheses without systematic pre-stenting in selected large native RVOTs and early experience with the Myval Octacor platform in dysfunctional conduits and pulmonary bioprostheses [26,27]. Nevertheless, the short duration of follow-up precludes definitive assessment of frame stability, valve thrombosis, infective endocarditis, structural valve deterioration, and the need for subsequent pulmonary valve reintervention, which are particularly important in the younger congenital heart disease population.

A systematic review published in 2025 synthesized the initial experience with Myval across aortic, mitral, tricuspid, and pulmonary indications, but its literature search extended only through May 2024 and was weighted predominantly toward aortic implantation [28]. Since that search, additional evidence has emerged regarding extra-large valves in native RVOTs, Myval Octacor implantation in the pulmonary position, ViMAC, and technically modified mitral delivery strategies [17,18,26,27]. A dedicated contemporary evaluation of non-aortic applications is therefore warranted. Importantly, the current evidence remains heterogeneous and is characterized by small sample sizes, retrospective designs, overlapping populations, variable outcome definitions, limited independent adjudication, and a scarcity of comparative or long-term data.

**Figure 1:**
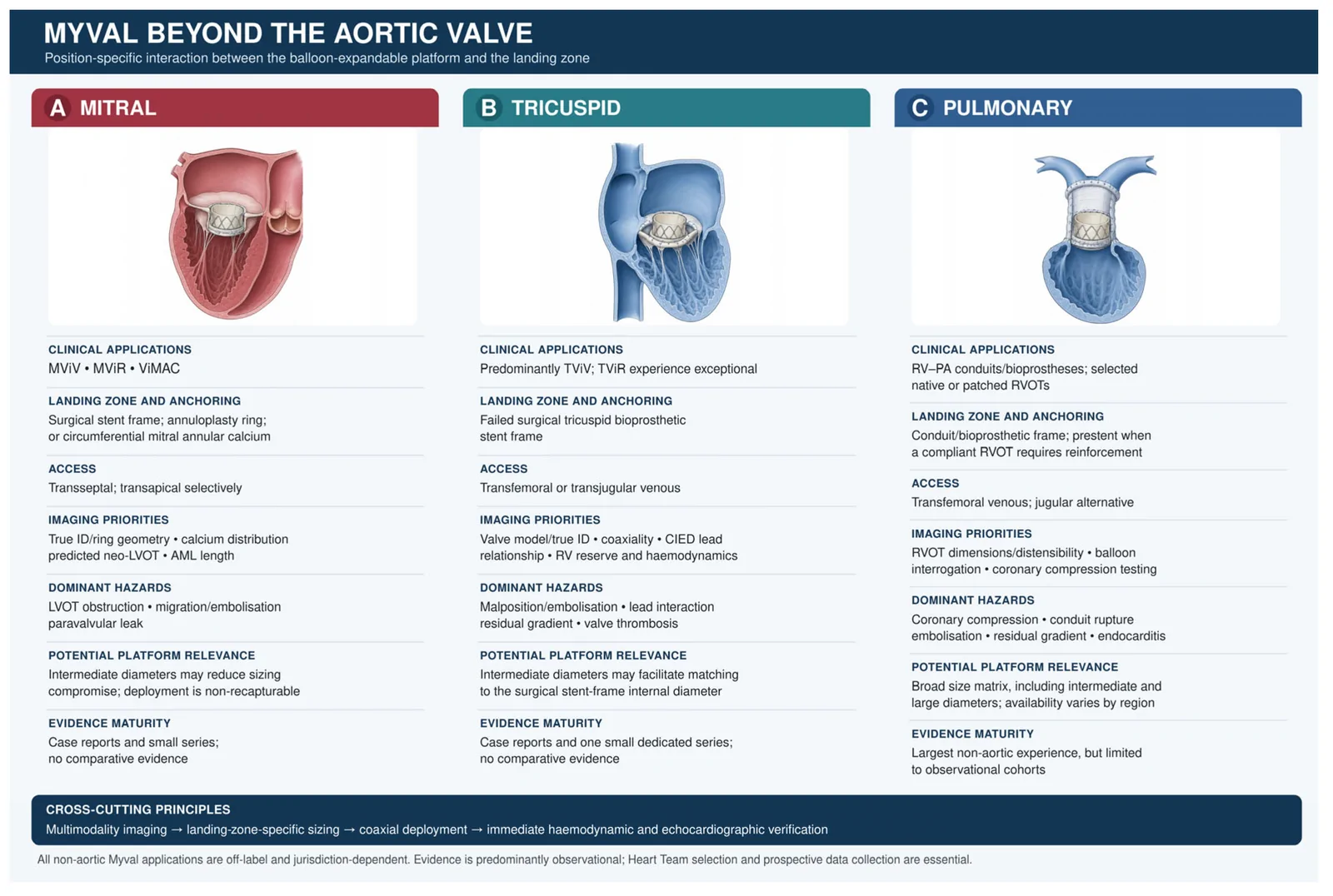
Position-specific device–landing-zone interactions during non-aortic Myval implantation. (**A**) Mitral applications include valve-in-valve, valve-in-ring, and valve-in-mitral annular calcification, requiring assessment of anchoring, landing-zone geometry, and neo-left ventricular outflow tract obstruction. (**B**) Tricuspid implantation requires evaluation of prosthesis or ring geometry, venous trajectory, device-lead interaction, and right-ventricular function. (**C**) Pulmonary implantation requires assessment of right ventricular outflow tract geometry, distensibility, and coronary-compression risk. Intermediate sizes increase nominal sizing granularity, but their effect on anchoring, sealing, residual gradients, and clinical outcomes has not been established; deployment is non-recapturable. All applications are off-label and supported predominantly by observational evidence. Abbreviations: AML, anterior mitral leaflet; CIED, cardiac implantable electronic device; ID, internal diameter; LVOT, left ventricular outflow tract; MViR, mitral valve-in-ring; MViV, mitral valve-in-valve; neo-LVOT, predicted residual left ventricular outflow tract after transcatheter mitral valve replacement; RV, right ventricle; RV–PA, right ventricle-to-pulmonary artery; RVOT, right ventricular outflow tract; TViR, tricuspid valve-in-ring; TViV, tricuspid valve-in-valve; ViMAC, valve-in-mitral annular calcification.

Accordingly, this review aims to critically evaluate the use of the Myval THV series in mitral, tricuspid, and pulmonary positions. The review will examine the relationship between device characteristics and landing-zone anatomy; summarize patient selection, multimodality imaging, sizing, access, deployment, and adjunctive procedural strategies; synthesize the available clinical and hemodynamic outcomes; and identify the principal unresolved questions concerning anchoring, left ventricular outflow tract obstruction, paravalvular regurgitation, coronary compression, valve thrombosis, infective endocarditis, and long-term durability. The aim is to distinguish supported clinical observations from procedural extrapolation and case-based hypotheses. The principal position-specific anatomical substrates and device–landing-zone interactions relevant to non-aortic Myval implantation are summarized in Figure 1.

## 2. The Myval Platform

### 2.1. Bioprosthesis Architecture

The Myval transcatheter heart valve (Meril Life Sciences, Vapi, India) is a balloon-expandable bioprosthesis originally engineered for transcatheter aortic valve implantation. The prosthesis comprises three decellularized bovine-pericardial leaflets mounted within a radiopaque nickel–cobalt alloy frame (MP35N). The first-generation frame has a hybrid honeycomb configuration: its upper portion consists of a single row of relatively large open hexagonal cells, whereas the lower portion contains two rows of more densely arranged closed cells. This configuration was designed to combine frame flexibility and access through the upper cells with greater radial strength at the intended annular anchoring segment [9].

The lower, or inflow, portion of the frame is covered internally by a polyethylene terephthalate (PET) sealing cuff. An additional external PET skirt surrounds the lowest cell row and is intended to reduce paravalvular regurgitation by occupying small residual spaces between the expanded frame and the surrounding landing zone [9]. Although this sealing system may be advantageous within relatively circular surgical bioprostheses or conduits, it cannot compensate for major geometric discontinuities, extensive ring dehiscence, or incomplete circumferential contact in irregular mitral annular calcification. Consequently, adequate mechanical support from the target anatomy remains essential.

### 2.2. Size Matrix and Delivery System

A distinctive characteristic of the Myval platform is its graduated size matrix. In addition to conventional nominal diameters of 20, 23, 26, and 29 mm, the platform includes intermediate sizes of 21.5, 24.5, and 27.5 mm and extra-large sizes of 30.5 and 32 mm [12,29]. In aortic practice, intermediate sizes were selected in 42% of 1115 Myval procedures, indicating that operators frequently encounter measurements falling between conventional device categories [29]. However, that study evaluated size-selection patterns rather than size-specific clinical outcomes.

In non-aortic applications, the narrower nominal increments provide additional sizing options when the measured landing zone lies between conventional valve diameters. This may reduce the numerical discrepancy between the selected prosthesis and the true internal diameter of a surgical valve, ring, conduit, or prestented right ventricular outflow tract. Nevertheless, nominal diameter alone does not determine anchoring, sealing, or valve expansion, which also depend on landing-zone geometry, rigidity, calcification, tissue ingrowth, circumferential support, implantation depth, and balloon-filling strategy. No comparative non-aortic study has demonstrated that intermediate sizing reduces embolization, migration, paravalvular regurgitation, residual gradient, frame underexpansion, thrombosis, or reintervention. Intermediate-size availability should therefore be described as increased sizing granularity rather than an established clinical advantage.

The valve is manually crimped onto the proprietary over-the-wire Navigator balloon catheter using a dedicated mechanical crimper. The Navigator system incorporates a flexion segment and two opposing soft stoppers that define the crimping zone and limit longitudinal valve displacement during delivery. The balloon is designed to begin expansion at its extremities, producing a transient dog-bone configuration that stabilizes the prosthesis during deployment. The established 20–32 mm devices are compatible with the expandable 14-Fr Python introducer sheath [9]. These features permit a relatively controlled, single-step implantation; nevertheless, as with other balloon-expandable prostheses, meaningful repositioning or recapture is not possible once balloon inflation has commenced. Precise fluoroscopic and echocardiographic alignment before deployment is therefore critical, particularly in short surgical valves, non-circular rings, and landing zones with limited axial support.

### 2.3. Myval Octacor

Myval Octacor is the second-generation balloon-expandable platform. It replaces the first-generation three-row hybrid hexagonal frame with two rows of interlacing octagonal cells while retaining the nine-diameter 20–32 mm size matrix. Its principal modifications include an enlarged internal polyethylene terephthalate cuff, a higher external sealing skirt, a radiopaque landing-zone marker, and lower reported deployment foreshortening of approximately 19–20%, compared with 21–24% for the first-generation valve. Octacor is delivered using the Navigator Inception system through the expandable 14-Fr Python sheath [30,31,32]. These modifications may improve depth control and sealing in selected non-aortic landing zones, but position-specific clinical evidence remains limited and does not establish superiority over the first-generation platform.

### 2.4. Myval Octapro

Myval Octapro is the third and most recent iteration of the balloon-expandable Myval platform. It retains an intra-annular trileaflet bovine-pericardial valve mounted within a cobalt-alloy frame but introduces a two-row hybrid-cell architecture. The outflow zone, comprising approximately 55% of the total frame height, consists of V-shaped chevrons formed by alternating upright and inverted crowns, whereas the inflow zone comprises interlacing octagonal cells. Total frame height ranges from 19.5 to 23.5 mm across the available sizes. This configuration has been associated with reported deployment foreshortening of approximately 10–12%, compared with 19–20% for Octacor and 21–24% for the first-generation Myval. Octapro also incorporates an internal PET sealing skirt and a higher external skirt than the first-generation device. It retains the nine-diameter 20–32 mm size matrix and is delivered using the Navigator Pro system through the expandable 14-Fr Python sheath [33].

**Figure 2:**
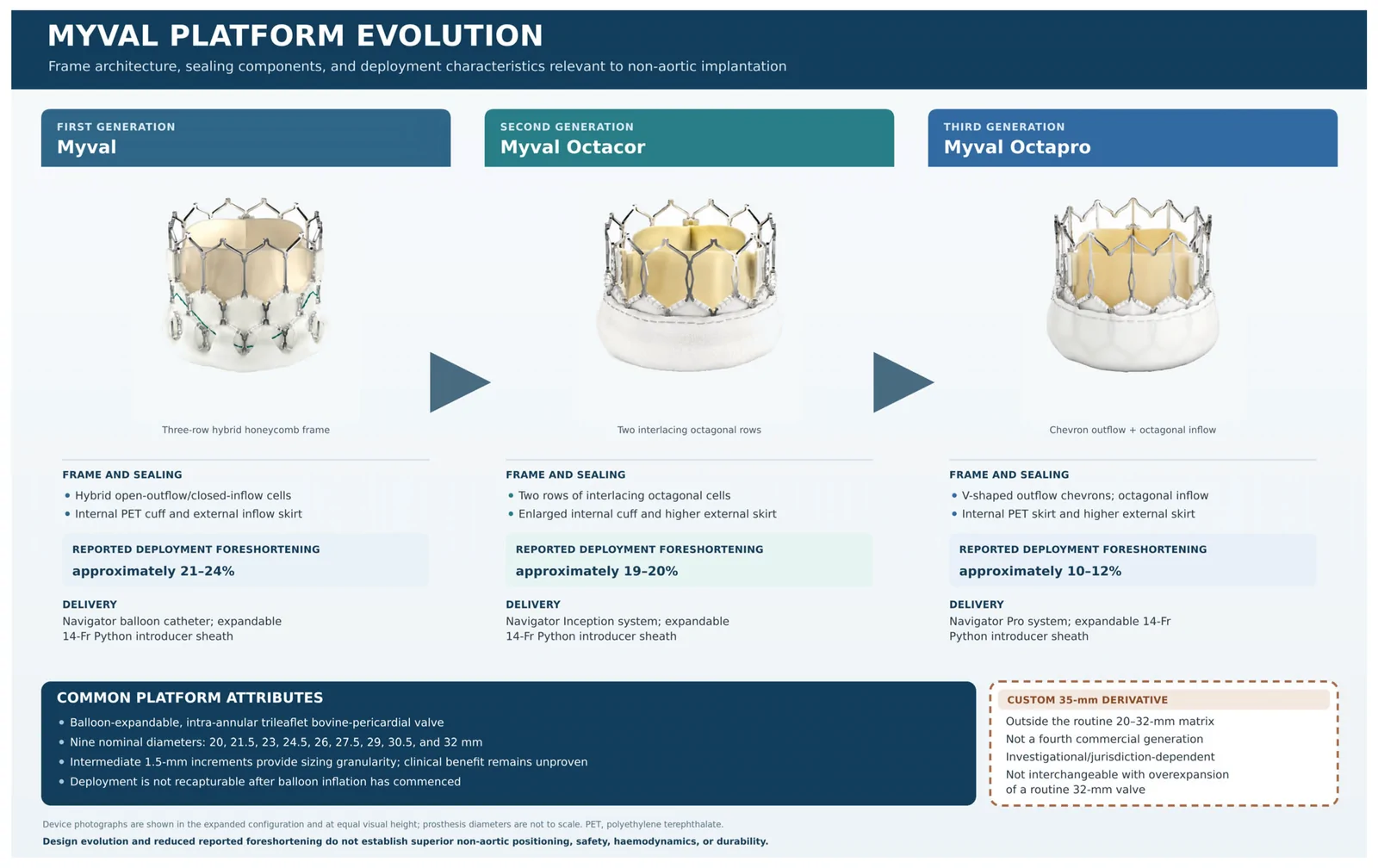
Evolution of the Myval balloon-expandable transcatheter heart valve platform. The figure compares the first-generation Myval, Myval Octacor, and Myval Octapro with respect to frame architecture, sealing components, delivery system, and reported deployment foreshortening. All three platforms retain an intra-annular trileaflet bovine pericardial valve and the nine-diameter 20–32 mm size matrix. The custom-manufactured nominal 35 mm Myval derivative is identified separately because it lies outside the routine platform and should not be regarded as a distinct commercial generation. Device photographs are displayed in the expanded configuration and at similar visual height; prosthesis diameters are not shown to scale. The figure illustrates design evolution and does not imply comparative clinical superiority. Images reproduced with permission from Meril Life Sciences.

Reduced deployment foreshortening may increase agreement between the intended and achieved axial frame position when the acceptable implantation-depth window is narrow. The most plausible non-aortic rationale would be in selected mitral valve-in-valve or complete, rigid valve-in-ring procedures with secure circumferential anchoring, in which small deviations toward the ventricle or atrium could, respectively, increase left ventricular outflow tract or subvalvular interaction or compromise frame engagement and sealing. However, reduced foreshortening should not be equated with a shorter implanted prosthesis or inherent suitability for a short landing zone. Because Octapro retains more of its axial height during expansion, it may increase atrial, ventricular, or vascular protrusion beyond the landing zone. Planning should therefore consider generation-specific final frame height, skirt position, fluoroscopic landmarks, and clearance from adjacent structures rather than foreshortening percentage alone. No comparative non-aortic study has demonstrated that Octapro improves implantation-depth accuracy or reduces migration, embolization, paravalvular leakage, residual gradients, left ventricular outflow tract obstruction, or reintervention. Octapro may therefore be considered when preprocedural imaging identifies a narrow safe depth window and adequate anchoring, but it cannot presently be recommended preferentially over Octacor or first-generation Myval. Figure 2 illustrates the evolution of frame geometry and inflow sealing components across the first-generation Myval, Octacor, and Octapro platforms; generation-specific technical attributes relevant to non-aortic implantation are summarized in Table 1.

### 2.5. Device Characteristics Relevant to Non-Aortic Implantation

Several properties of the Myval platform provide a mechanistic rationale for its use beyond the aortic position: a broad size spectrum, high balloon-generated radial force, radiopaque frame geometry, relatively low-profile delivery, and rapid, controlled expansion. These characteristics may be particularly useful in valve-in-valve and selected valve-in-ring procedures, where a pre-existing surgical frame provides a defined landing zone [13,14,15,16,19,20,21,22,23,24,25]. Nevertheless, Myval has no active fixation mechanism, atrial flange, ventricular anchors, or dedicated tethering system. Procedural stability therefore depends on radial interaction with the surgical prosthesis, ring, calcification, conduit, or prestent.

The intended direction of transvalvular flow and the selected access route must also be considered when the valve is crimped, because non-aortic implantation may require an orientation opposite to that used during retrograde transfemoral aortic delivery. Frame height, expected foreshortening, and implantation depth should be assessed in relation to adjacent structures, including the left ventricular outflow tract, circumflex coronary artery, pulmonary arteries, and right ventricular outflow tract bifurcation. Excessive oversizing may improve anchoring but can produce frame underexpansion, leaflet pinwheeling, elevated gradients, annular injury, or uncertain long-term durability; insufficient oversizing may result in migration or paravalvular regurgitation. Accordingly, the platform’s dimensional versatility should be regarded as an additional planning option rather than evidence of intrinsic suitability for every non-aortic anatomy. Current experience supports technical feasibility in carefully selected patients, but dedicated bench testing, standardized sizing algorithms, and prospective non-aortic studies remain necessary [28].

## 3. Device–Landing-Zone Interaction

### 3.1. General Biomechanical Principles

The performance of a balloon-expandable transcatheter heart valve is determined by the interaction between the device and its recipient landing zone rather than by either component in isolation. An adequate landing zone must provide three interrelated functions: radial containment of the expanded frame, resistance to axial migration, and sufficiently continuous circumferential contact to achieve sealing. These functions should be evaluated separately, because a structure may provide effective anchoring but incomplete sealing, or conversely appear well sealed immediately after implantation while offering insufficient resistance to subsequent migration.

Myval does not incorporate active fixation mechanisms such as hooks, ventricular anchors, atrial flanges, or tethers. Its stability therefore depends predominantly on balloon-generated radial expansion, friction against the surrounding substrate, geometric constraint, and appropriate oversizing. The magnitude and distribution of these forces vary according to the rigidity, shape, continuity, and compliance of the recipient structure. Undersizing reduces radial contact and increases the risk of migration and paravalvular regurgitation, whereas excessive oversizing may cause incomplete frame expansion, distortion, injury to the surrounding tissue, or abnormal leaflet configuration. Experimental evidence indicates that transcatheter valve underexpansion can induce leaflet pinwheeling and substantially increase leaflet stresses, providing a plausible mechanism for impaired durability [34]. Accordingly, achieving a stable implant should not be equated with maximizing oversizing.

### 3.2. Surgical Bioprosthetic Valves

Failed stented surgical bioprostheses generally represent the most predictable non-aortic landing zones. Their sewing ring and stent frame provide a predefined, usually circular and circumferentially continuous scaffold capable of constraining the transcatheter prosthesis. Nevertheless, the labeled surgical valve size does not correspond reliably to the available lumen. The relevant parameter is the true internal diameter, defined by the narrowest functional diameter after accounting for the surgical frame, leaflet mounting, fabric covering, pannus, calcification, and structural degeneration [8,35]. Direct review of the operative report, valve card, fluoroscopic appearance, manufacturer specifications, and gated computed tomography should therefore be integrated whenever possible.

Anchoring occurs predominantly where the expanded transcatheter frame interacts with the surgical sewing ring or narrowest stent segment. Excessively atrial or ventricular deployment may reduce the length of frame engagement, compromise sealing, or expose the valve to axial displacement. Conversely, implantation within a small true internal diameter can produce substantial frame underexpansion and elevated residual gradients. The intermediate Myval sizes may permit closer matching between the transcatheter valve and surgical true internal diameter, potentially reducing the need for marked over- or undersizing; however, this proposed advantage has not been tested comparatively in non-aortic valve-in-valve procedures [13,14,15,16,20,21,22,23,24].

The relatively reproducible geometry of a stented surgical valve partly explains why mitral, tricuspid, and pulmonary valve-in-valve procedures have generally achieved higher technical success than interventions involving annuloplasty rings or native calcified annuli [5,6,7]. Nevertheless, degeneration-related asymmetry, pannus, leaflet calcification, surgical-valve deformation, and pre-existing paravalvular dehiscence may convert an apparently circular prosthesis into an irregular or incomplete landing zone.

### 3.3. Annuloplasty Rings

Annuloplasty rings constitute a heterogeneous group of landing zones characterized by important differences in completeness, rigidity, three-dimensional configuration, radiopacity, and capacity for circularization. These properties are more relevant to transcatheter valve implantation than the manufacturer’s labeled ring size. Complete semirigid rings generally provide the most favorable balance between circumferential support and the ability to accommodate a circular balloon-expandable frame. Rigid rings may provide strong fixation but resist circularization, resulting in elliptical valve expansion, incomplete apposition, paravalvular leakage, and potentially abnormal leaflet mechanics. Highly flexible rings may become circular but provide insufficient radial counterforce, whereas incomplete bands lack anterior support and may permit both paravalvular regurgitation and device migration [8].

Because most mitral and tricuspid annuloplasty rings are D-shaped, saddle-shaped, or otherwise non-circular, sizing based on a single diameter is inadequate. Ring area, perimeter, minimum and maximum diameters, internal material, and predicted circularized diameter should be assessed. Balloon expansion may alter the geometry of deformable rings, meaning that post-implantation dimensions may differ substantially from baseline measurements. The Myval external skirt may improve sealing across small residual channels but cannot reliably bridge a large unsupported segment or compensate for ring dehiscence. These anatomical limitations are consistent with the lower technical success and higher rates of residual regurgitation and repeat valve implantation reported after mitral valve-in-ring compared with valve-in-valve procedures [5,8,15,19].

### 3.4. Mitral Annular Calcification

In valve-in-MAC procedures, the landing zone is formed by native calcium rather than a manufactured circular scaffold. MAC is frequently eccentric, nodular, discontinuous, and concentrated posteriorly, whereas the anterior annulus and fibrous trigones may remain relatively unsupported. Consequently, severe total calcium burden does not necessarily indicate a mechanically adequate landing zone. The distribution, circumferential extent, thickness, and continuity of calcium are more relevant to anchoring than calcium volume alone.

The CT-based MAC score incorporates average calcium thickness, circumferential involvement, calcification of the fibrous trigones, and leaflet involvement and has been associated with the risk of valve embolization or migration during transseptal valve-in-MAC procedures [36]. Limited or discontinuous calcification may provide insufficient fixation, while extensive circumferential calcium improves anchoring but may restrict frame expansion and increase the risk of annular injury. Balloon expansion against rigid nodular deposits may also produce asymmetric frame deformation, residual paravalvular channels, or displacement of calcium toward adjacent myocardium.

Anchoring and left ventricular outflow tract obstruction must be considered simultaneously. A more ventricular implant may increase interaction with the calcified annulus but also displace the anterior mitral leaflet and prosthetic frame toward the interventricular septum, reducing the neo-left ventricular outflow tract. Conversely, an excessively atrial position may reduce annular engagement and increase migration risk. Thus, the selected implantation depth represents a compromise between retention, sealing, leaflet function, and preservation of the outflow tract [4,5,17,18].

### 3.5. Right-Sided Landing Zones

Tricuspid surgical bioprostheses generally provide a circular and continuous landing zone similar to mitral bioprostheses, whereas tricuspid annuloplasty rings may be incomplete, large, and geometrically irregular. Although right-sided pressure loads are lower, inadequate circumferential support may still result in malposition, paravalvular regurgitation, or delayed migration [7,19,20,21].

In the pulmonary position, conduits and stented surgical bioprostheses provide relatively defined landing zones. Calcified conduits may supply strong radial constraint but carry risks of rupture and non-uniform expansion. Prestenting can create a more cylindrical scaffold, reduce recoil, and increase the axial length available for valve fixation. In contrast, native or transannular-patch RVOTs are often large, compliant, tapered, and dynamically variable throughout the cardiac cycle. Multiphase CT assessment should therefore include minimum and maximum diameters, perimeter, cross-sectional area, landing-zone length, RVOT–pulmonary artery transition, and relationships with the coronary arteries and aortic root [37]. Balloon interrogation may further identify a reproducible waist and exclude coronary or aortic compression.

The larger Myval sizes expand the range of potentially treatable RVOT dimensions, but valve diameter alone cannot overcome the absence of a stable circumferential landing zone. Prestenting, staged remodeling, or hybrid surgical reduction may remain necessary in highly compliant or aneurysmal RVOTs [23,24,25,26,27]. Across all non-aortic positions, procedural suitability should therefore be defined by the predicted behavior of the combined device–landing-zone system: stable axial retention, adequate sealing, acceptable frame expansion, unobstructed adjacent structures, and preserved leaflet function [28]. These position-specific measurements, failure modes, quantitative risk markers, and procedural safeguards are summarized in Table 2.

### 3.6. Positioning Relative to Dedicated Native-Valve Replacement Systems

The distinction between implantation within a pre-existing landing zone and replacement of a non-calcified native valve is fundamental. Myval depends on radial expansion, friction, and geometric constraint and does not incorporate active annular, leaflet, or ventricular fixation. Dedicated native mitral and tricuspid replacement systems should therefore be preferred when the annulus is large, compliant, markedly non-circular, or dynamically variable and lacks a rigid circumferential substrate. Position-specific devices may incorporate annular or leaflet anchors, atrial flanges, docking structures, or apical tethering and may offer repositionability or recapture, although these characteristics and their clinical evidence vary among platforms. Their use remains dependent on device-specific anatomical eligibility, access, ventricular reserve, anticipated clinical benefit, and jurisdictional availability [2].

In the native mitral position, direct Myval implantation should be confined to selected ViMAC anatomies in which sufficiently extensive and continuous calcium provides credible anchoring and sealing. A CT MAC score ≥ 7 is associated with a lower risk of migration or embolization than lower scores but does not independently establish eligibility [36]. Annular dimensions must permit nominal valve deployment, while predicted neo-LVOT, implantation depth, anterior leaflet interaction, annular-injury risk, and anticipated paravalvular regurgitation must remain acceptable [38,39]. A dedicated mitral replacement system should be preferred for non-calcified native mitral disease, insufficient or discontinuous MAC, or anatomy requiring active fixation or a conformable sealing frame. Myval may be considered when a dedicated device is unavailable or unsuitable for device-specific reasons only if the independent anatomical requirements for Myval implantation are fully satisfied.

For native tricuspid replacement, a dedicated orthotopic system should be preferred because the non-calcified tricuspid annulus generally cannot provide predictable fixation for Myval. Current Myval evidence is limited to implantation within failed surgical bioprostheses and selected annuloplasty rings [7,19,20,21,22]. Accordingly, Myval is a reasonable option for TViV and carefully selected TViR, but not for direct implantation within an unsupported native tricuspid annulus. Implantation after intentional creation of a docking scaffold should be considered investigational. Importantly, failure to meet the eligibility criteria for a dedicated device does not create eligibility for Myval when the reason for exclusion is absent anchoring, prohibitive adjacent-structure interaction, advanced ventricular dysfunction, severe pulmonary vascular disease, or limited anticipated clinical benefit.

### 3.7. Proposed Anatomy-First Decision Framework

Quantitative risk thresholds have been established for only selected aspects of non-aortic transcatheter valve planning. In ViMAC, a CT-based MAC score ≤ 6 identifies an increased risk of valve migration or embolization [36]. In pulmonary planning, a coronary-to-landing-zone distance ≤ 3 mm and a landing-zone-to-chest-wall distance ≤8 mm indicate increased coronary-compression risk, although dynamic balloon interrogation remains decisive [37]. A predicted end-systolic neo-LVOT area ≤ 170 mm^2^ identifies increased LVOT-obstruction risk after balloon-expandable TMVR [38,39]. In contrast, no validated cut-offs exist for ring eccentricity, the maximum tolerable unsupported arc, minimum supported circumference, or minimum axial landing-zone length. These measurements should therefore be reported as continuous variables and interpreted within the complete device–anatomy interaction rather than used as isolated eligibility criteria.

**Figure 3:**
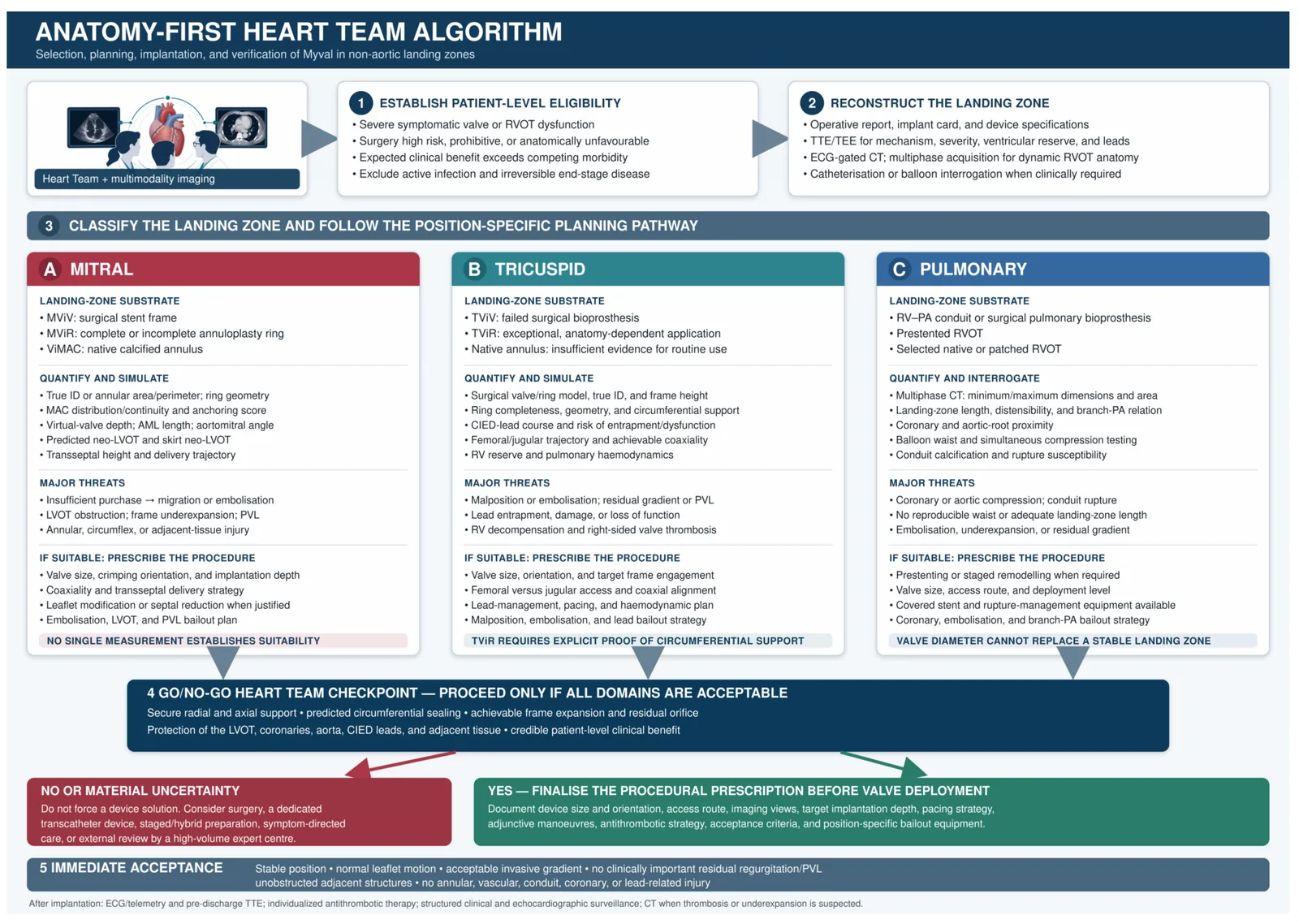
Anatomy-first Heart Team algorithm for non-aortic Myval implantation. Multimodality imaging and, when indicated, balloon interrogation are integrated to assess landing-zone geometry, anchoring, sealing, access trajectory, and interaction with adjacent structures. The framework represents a synthesis of published evidence and expert opinion and has not been prospectively validated. Myval-specific evidence is lacking for the sequence and weighting of its branches, anchoring and sealing thresholds, sizing and platform selection, implantation depth, indications for adjunctive procedures, position-specific ventricular criteria, and post-implantation acceptance thresholds. It should not be interpreted as a clinical prediction rule or stand-alone treatment recommendation. All non-aortic Myval applications are off-label and jurisdiction-dependent. Abbreviations: AML, anterior mitral leaflet; CIED, cardiac implantable electronic device; CT, computed tomography; ID, internal diameter; LVOT, left ventricular outflow tract; MAC, mitral annular calcification; MViR, mitral valve-in-ring; MViV, mitral valve-in-valve; neo-LVOT, predicted residual left ventricular outflow tract after transcatheter mitral valve replacement; PA, pulmonary artery; PVL, paravalvular leak; RV, right ventricle; RV–PA, right ventricle-to-pulmonary artery; RVOT, right ventricular outflow tract; TEE, transoesophageal echocardiography; TTE, transthoracic echocardiography; TViR, tricuspid valve-in-ring; TViV, tricuspid valve-in-valve; ViMAC, valve-in-mitral annular calcification.

Figure 3 integrates these anatomical and procedural considerations into a proposed Heart Team decision framework. It represents a synthesis of published evidence and expert opinion and has not been prospectively validated. Although several individual measurements are supported by broader transcatheter valve experience, their sequence, relative weighting, and Myval-specific application remain untested. Evidence is particularly limited regarding the minimum substrate required for anchoring and sealing, intermediate-size selection, oversizing, choice among Myval generations, optimal implantation depth, indications for adjunctive leaflet, septal, prestenting, or hybrid techniques, tricuspid right-ventricular and lead-related selection criteria, pulmonary landing-zone dimensions, and post-implantation acceptance thresholds. The framework is intended to structure multidisciplinary assessment and should not be used as a stand-alone indication for Myval implantation.

**Table 2:** Device–landing-zone interaction: position-specific measurements, failure modes, and procedural safeguards.

Landing-Zone Substrate	Mechanical Behavior and Anchoring	Measurements That Drive Sizing	Dominant Failure Modes	Risk-Mitigation Strategy	Quantitative High-Risk/No-Go Features and Evidence Limitations
Stented surgical bioprosthesis MViV, TViV, pulmonary ViV	Usually the most reproducible substrate: a pre-existing stent frame and sewing ring provide circumferential radial support. Pannus, leaflet bulk, and internal frame design may reduce the functional lumen.	Identify exact valve model and label size; use true internal diameter rather than label diameter; integrate CT-measured lumen, pannus, and calcific degeneration.	Malposition in short frames; underexpansion and high residual gradient; embolization if undersized; leaflet pinwheeling; coronary interaction in selected pulmonary/aortic-conduit geometries.	Confirm fluoroscopic landmarks; simulate final depth; choose the smallest size that provides secure fixation without injurious oversizing; consider balloon interrogation when the true lumen is uncertain.	The manufacturer-specific true internal diameter is mandatory; no universal minimum true internal diameter or oversizing threshold applies across positions. High-risk features include major prosthetic deformation or dehiscence and inability to obtain stable frame engagement without marked underexpansion or non-nominal overexpansion.
Complete semirigid annuloplasty ring MViR or TViR	May circularize under balloon expansion while providing circumferential support; final geometry depends on ring model, rigidity, and implantation integrity.	Ring model/label size; internal area, perimeter, minimum and maximum diameters; predicted circularized diameter; relation to adjacent myocardium and leaflets.	PVL from geometric mismatch; ring deformation or fracture; frame underexpansion; malposition; LVOT obstruction in MViR; RCA or conduction-system interaction in TViR.	Bench/app-based compatibility should be confirmed against CT. Plan implant depth and sealing zone; consider staged PVL closure only if a discrete, accessible residual channel is anticipated.	For MViR, a predicted end-systolic neo-LVOT ≤ 170 mm^2^ indicates high LVOT-obstruction risk. No validated ring–THV area-, perimeter-, or apposition-mismatch threshold exists. Major dehiscence, broad predicted non-apposition, or unmitigable LVOT-obstruction risk constitutes a no-go.
Rigid or markedly non-circular complete ring	Limited circularization concentrates contact and may leave unsupported commissural gaps; radial force may distort either the ring or THV frame.	Multiplanar CT/3D echocardiography; eccentricity; predicted frame deformation; circumferential apposition; device-to-ring area and perimeter mismatch.	Persistent PVL, high gradient, leaflet distortion, frame recoil, ring injury, or embolization.	Avoid aggressive oversizing as a substitute for apposition. Predefine bailout options: second valve, plug closure, or surgery.	No validated eccentricity or supported-circumference threshold exists. Report minimum and maximum diameters, their ratio, predicted frame eccentricity, and apposed arc in degrees and percentage of circumference. Predicted major frame distortion or absence of secure anchoring and sealing without a feasible bailout strategy constitutes a no-go.
Incomplete ring or band	The open segment lacks circumferential counterforce; the THV may expand preferentially toward the unsupported zone. The external skirt cannot bridge a large structural gap.	Ring completeness and open-segment arc; dehiscence; unsupported tissue quality; minimum/maximum diameters; anticipated plug-access trajectory.	Severe PVL through the open segment, instability, migration, hemolysis, or need for secondary closure.	Treat only after explicit demonstration of adequate anchoring elsewhere; plan intraprocedural TEE and a rescue strategy. A discrete residual PVL may be closed, but this should not be assumed.	No validated maximum tolerable unsupported arc or minimum supported circumference exists. Report the open segment in degrees and percentage of circumference and any dehiscence in millimetres. Broad non-apposition, progressive dehiscence, or absence of adequate radial purchase opposite the unsupported segment indicates high or prohibitive risk. Oversizing should not be assumed to compensate for absent support.
Severe native MAC ViMAC	Anchoring depends on the distribution, continuity, and thickness of calcium rather than nominal annular size. The landing zone is irregular and adjacent to the AML, circumflex artery, and LVOT.	MAC score and 3D calcium distribution; annular area/perimeter; trigone involvement; leaflet length; aortomitral angle; simultaneous virtual-valve neo-LVOT and skirt neo-LVOT.	Embolization, PVL, annular injury, LVOT obstruction, circumflex injury, incomplete expansion, and early or delayed migration.	Use CT simulation; define implantation depth and leaflet-modification/septal-reduction strategy when required; consider external support or hybrid stabilization only in expert centers.	A CT MAC score ≤ 6 is high risk: migration/embolization occurred in 60% versus 9.7% with scores ≥ 7. Circumferential calcium > 270° contributes the maximum circumference component but is not independently sufficient for anchoring. Predicted end-systolic neo-LVOT ≤ 170 mm^2^ indicates high LVOT-obstruction risk. Unmitigable anchoring or LVOT risk constitutes a no-go.
Calcified RV–PA conduit or prestented RVOT	A conduit or prestent can provide a relatively cylindrical landing zone. Calcification increases fixation but may reduce compliance and increase rupture risk.	Multiphase CT and angiography: narrowest diameter, conduit length, calcification, branch PA relation, coronary proximity; balloon waist and occlusion testing.	Conduit rupture, underexpansion, stent fracture, coronary compression, embolization, residual gradient, or PR.	Prestent when needed to create an adequate landing cylinder; perform simultaneous coronary angiography during balloon interrogation; keep covered stent and rupture-management equipment available.	Coronary-to-landing-zone distance ≤ 3 mm or landing-zone-to-chest-wall distance ≤ 8 mm indicates increased coronary-compression risk. A positive coronary- or aortic-compression test is a no-go. Heavy conduit calcification increases rupture risk, but no validated Myval-specific calcium-burden or conduit-expansion threshold exists. A required diameter outside the routine 20–32 mm matrix necessitates an investigational rather than routine strategy.
Native or patch-enlarged RVOT	Often compliant, tapered, aneurysmal, elliptical, and dynamic; the narrowest functional landing zone may differ across the cardiac cycle. Fixation may be absent without prestenting.	Multiphase CT/CMR; systolic and diastolic diameters, area/perimeter, length, taper and aneurysms; balloon waist/occlusion; simultaneous coronary and aortic-root assessment.	Migration, asymmetric expansion, residual PR, coronary compression, branch-PA obstruction, RVOT injury, arrhythmia, or late frame instability.	Select direct implantation only when a stable reproducible waist and adequate axial length are demonstrated. Prestent unstable or aneurysmal zones. Repeat coronary assessment if the final valve diameter exceeds the test balloon.	The same CT proximity thresholds—≤3 mm to a coronary artery and ≤8 mm to the chest wall—indicate increased compression risk [37]. No reproducible balloon waist, failure to achieve balloon occlusion, positive coronary/aortic compression, or absence of adequate axial purchase constitutes a no-go. No validated Myval-specific threshold exists for dynamic diameter change or minimum landing-zone length; a required diameter > 32 mm is investigational.
Non-calcified native mitral or tricuspid annulus	Large, compliant, non-circular, and dynamically variable; no surgical frame or annular calcium provides radial constraint. Myval has no active fixation mechanism.	Multiphase annular area, perimeter, minimum and maximum diameters, dynamic change, leaflet and subvalvular anatomy; neo-LVOT and skirt neo-LVOT for mitral replacement; RV function, pulmonary pressure, lead interaction, and adjacent coronary anatomy for tricuspid replacement.	Migration or embolization, frame malapposition, severe paravalvular regurgitation, asymmetric expansion, leaflet or subvalvular interaction, LVOT obstruction in the mitral position, and acute RV afterload intolerance in the tricuspid position.	Prefer a dedicated native-valve replacement system when replacement is appropriate, or consider repair, surgery, or a clinical trial. Myval requires a pre-existing or intentionally created rigid landing scaffold.	Absence of a rigid, sufficiently circumferential landing zone constitutes a no-go for direct Myval implantation. No validated strategy supports compensating for absent fixation through oversizing alone. Exclusion from a dedicated-device program does not independently establish Myval eligibility.

Abbreviations: AML, anterior mitral leaflet; CMR, cardiovascular magnetic resonance; CT, computed tomography; LVOT, left ventricular outflow tract; MAC, mitral annular calcification; MViR, mitral valve-in-ring; MViV, mitral valve-in-valve; PA, pulmonary artery; PR, pulmonary regurgitation; PVL, paravalvular leak; RCA, right coronary artery; RVOT, right ventricular outflow tract; TEE, transoesophageal echocardiography; THV, transcatheter heart valve; TViR, tricuspid valve-in-ring; TViV, tricuspid valve-in-valve; ViMAC, valve-in-MAC. Quantitative thresholds in this table are risk-stratification markers derived predominantly from studies of other balloon-expandable platforms and have not been validated specifically for Myval. The CT MAC score assigns points for average calcium thickness (<5 mm, 1 point; 5–9.9 mm, 2 points; ≥10 mm, 3 points), circumferential involvement (<180°, 1 point; 180–270°, 2 points; >270°, 3 points), fibrous-trigone involvement (0–2 points), and leaflet involvement (0–2 points); total scores ≤ 3, 4–6, and ≥7 denote mild, moderate, and severe MAC, respectively. Numerical thresholds should not be interpreted as isolated indications or contraindications. A no-go designation indicates that the anticipated risk cannot be adequately mitigated using an alternative device or size, leaflet or septal modification, prestenting, hybrid stabilization, or a credible bailout strategy.

## 4. Mitral Applications

Transcatheter mitral valve replacement using the Myval platform encompasses three anatomically and procedurally distinct applications: implantation within a failed surgical bioprosthesis (mitral valve-in-valve; MViV), within a previously implanted annuloplasty ring (mitral valve-in-ring; MViR), and within severe native mitral annular calcification (valve-in-MAC; ViMAC). Although these procedures share the use of a balloon-expandable transcatheter heart valve, their anchoring mechanisms, anatomical constraints, procedural predictability, and clinical risk differ substantially. Accordingly, evidence derived from one mitral application should not be extrapolated uncritically to another.

### 4.1. Clinical Indications and Anatomical Selection

Redo mitral valve surgery remains the reference treatment for failed surgical bioprostheses or annuloplasty repairs in patients with acceptable operative risk. Transcatheter implantation should therefore be considered principally in symptomatic patients with severe prosthetic dysfunction or recurrent mitral regurgitation who are judged by a multidisciplinary Heart Team to be at high or prohibitive surgical risk [2,3,5]. Patient selection should integrate life expectancy, frailty, comorbidities, right- and left-ventricular function, pulmonary hypertension, concomitant valvular disease, and the probability of obtaining a technically and hemodynamically satisfactory result.

Transthoracic and transoesophageal echocardiography define the mechanism and severity of valve failure, including stenosis, intraprosthetic regurgitation, paravalvular regurgitation, or mixed dysfunction. Isolated paravalvular leakage requires particular attention because implantation of a second valve may not correct a defect located outside the surgical sewing cuff; dedicated paravalvular closure or surgery may be more appropriate in selected cases. Active endocarditis, untreated intracardiac thrombus, inadequate annular anchoring, and a prohibitive predicted risk of left ventricular outflow tract obstruction (LVOTO) generally preclude transcatheter implantation.

Multidetector computed tomography is central to procedural planning. For MViV, the relevant dimension is the true internal diameter of the surgical prosthesis rather than its nominal label size, because leaflet mounting, stent design, pannus formation, and calcific degeneration may reduce the available lumen [35]. For MViR, ring completeness, rigidity, three-dimensional configuration, internal dimensions, and capacity for circularization must be assessed. ViMAC evaluation additionally requires characterization of the circumferential distribution, thickness, and continuity of annular calcium, for which CT-based MAC severity scores may assist in estimating the likelihood of anchoring, embolization, and paravalvular leakage [36].

Virtual implantation should reproduce the intended Myval size, ventricular depth, angulation, and expected frame expansion. The resulting neo-left ventricular outflow tract (neo-LVOT), and where appropriate the skirt neo-LVOT, should be assessed together with anterior mitral leaflet length, aortomitral angulation, septal thickness, left-ventricular dimensions, and baseline LVOT area [38]. In a historical balloon-expandable TMVR cohort, a predicted neo-LVOT area ≤1.7 cm^2^ was strongly associated with LVOTO; obstruction occurred substantially more frequently after ViMAC than after MViR or MViV and was associated with markedly increased procedural mortality [39]. This threshold should not be regarded as absolute, because its predictive performance depends on loading conditions, cardiac phase, implantation depth, device configuration, and the availability of preventive strategies.

### 4.2. Mitral Valve-in-Valve

MViV is the most standardized mitral application of balloon-expandable transcatheter valves. The circular stent frame and radiopaque sewing ring of a surgical bioprosthesis usually provide a definable landing zone, making sizing and deployment more predictable than in MViR or ViMAC. Nevertheless, small surgical valves, marked pannus, stent distortion, and prostheses with externally mounted or damaged leaflets may increase the risks of underexpansion, residual gradient, or acute leaflet dysfunction. Selection of the Myval size should therefore combine the identified prosthesis model, true internal diameter, CT measurements, and—when uncertainty persists—carefully performed balloon assessment [8,35].

The first dedicated multicenter Myval MViV study included 11 high-risk patients treated through a transseptal approach [13]. Technical success was achieved in all cases, and the mean transmitral gradient decreased from 14.7 ± 2.3 mmHg before intervention to 3.4 ± 1.7 mmHg after implantation. No intraprocedural death, valve migration, significant paravalvular leakage, or LVOTO was reported. At six months, one patient with suboptimal anticoagulation developed an increased gradient that normalized after optimization of anticoagulant therapy, illustrating the need to consider valve thrombosis when an otherwise unexplained gradient increase occurs.

A subsequent 20-patient series reported 100% technical success, without significant residual mitral regurgitation, paravalvular leakage, or LVOTO [16]. The postprocedural mean gradient was 4.6 ± 2.7 mmHg, while 30-day and one-year mortality were 5% and 10%, respectively. Surviving patients with available follow-up were in New York Heart Association functional class I or II. Interpretation should remain cautious because the cohort was small and only approximately half of the patients had completed one-year follow-up at the time of analysis.

The largest available multicenter experience included 97 Myval implantations in failed left-sided surgical valves or rings, of which 64 involved the mitral position [15]. Overall technical success was 98%, and survival in the mitral group was approximately 89% at a median follow-up of 15 months. Two mitral procedures developed acute transcatheter valve dysfunction requiring a second Myval: one after implantation within an annuloplasty ring and another in a degenerated surgical bioprosthesis whose protruding material appeared to interfere with the transcatheter leaflets. One patient developed delayed severe LVOTO, and another experienced valve thrombosis that resolved following conversion to warfarin. These events indicate that delayed anatomical interaction and leaflet thrombosis can occur despite technically successful deployment.

For context, a large registry of 1529 SAPIEN 3 MViV procedures reported 96.8% technical success, mortality of 5.4% at 30 days and 16.7% at one year, and a mean one-year gradient of 7.0 mmHg [40]. Transseptal implantation was associated with lower one-year mortality than transapical access. These findings establish a benchmark for balloon-expandable MViV but should not be interpreted as evidence of equivalence between valve platforms. Current Myval data remain derived from substantially smaller, non-randomized cohorts with limited longitudinal follow-up.

### 4.3. Mitral Valve-in-Ring

MViR is less predictable because surgical annuloplasty rings differ markedly in completeness, rigidity, radiopacity, saddle configuration, and ability to accommodate a circular transcatheter frame [5,8]. Complete or semirigid rings that can be circularized generally provide more favorable anchoring, whereas incomplete bands, highly rigid rings, markedly elliptical geometries, and very large rings may predispose to paravalvular regurgitation, underexpansion, malapposition, or embolization. Moreover, the retained anterior mitral leaflet introduces a greater potential for LVOTO than in most MViV procedures.

The Myval evidence base for MViR is limited predominantly to cases incorporated within mixed MViV/MViR cohorts, in which outcomes are not always reported separately [15,28]. The broad Myval size matrix may permit closer dimensional matching in selected rings, but additional nominal sizes do not compensate for an unsuitable landing-zone geometry. Oversizing intended to improve anchoring must be balanced against frame underexpansion, impaired leaflet motion, ring injury, and displacement toward the LVOT.

Evidence from other balloon-expandable platforms illustrates the residual risk of MViR. In a contemporary United States registry of 820 MViR procedures, technical success was 88.0%, while 30-day and one-year mortality were 8.3% and 22.4%, respectively [41]. The mean gradient at one year was 8.4 ± 3.4 mmHg, and reintervention occurred in 9.1% of patients. Although more than 90% had no more than mild residual mitral regurgitation, these outcomes remain less favorable and more heterogeneous than those reported for MViV.

When the predicted risk of LVOTO is unacceptable, modification of the anterior mitral leaflet may be considered. Tip-to-base LAMPOON followed by transseptal Myval implantation has been described in an individual MViR case [42]. This experience demonstrates technical compatibility but constitutes proof of concept rather than evidence supporting routine application.

### 4.4. Valve-in-Mitral Annular Calcification

ViMAC represents the most complex and highest-risk mitral application. In contrast to MViV, no surgically defined circular landing zone is present, and anchoring depends on the distribution and continuity of native annular calcium. Insufficient or eccentric calcification may result in inadequate fixation, migration, or severe paravalvular leakage, whereas extensive calcium may prevent symmetrical expansion and increase the risk of annular injury. Displacement of the anterior mitral leaflet and ventricular protrusion of the transcatheter frame also make LVOTO a central determinant of procedural eligibility [4,5,39,41].

Published Myval experience in ViMAC remains restricted to individual cases and very small series. In one report, an 84-year-old patient with severe circumferential MAC and a CT-based MAC score of 8 underwent transseptal implantation of a 30.5 mm Myval, without significant paravalvular leakage or LVOTO and with a mean transmitral gradient of 5 mmHg at six months [17]. Ninios et al. subsequently reported Myval implantation following balloon-assisted translocation of the mitral anterior leaflet (BATMAN) in a patient with circumferential MAC, a MAC score of 9, a 28 mm anterior mitral leaflet, and a predicted neo-LVOT area of only 80 mm^2^ [43]. Following electrosurgical leaflet perforation and balloon-mediated leaflet translocation, a 29 mm Myval was implanted through a transfemoral transseptal approach, with no residual transmitral or systolic LVOT gradient and no paravalvular leakage. A hybrid transseptal–transapical rail technique has additionally been used in three complex Myval mitral implantations, including one ViMAC procedure, with successful implantation and no reported embolization or LVOTO [18]. Although these reports provide important technical evidence regarding Myval implantation and adjunctive leaflet- or delivery-modification strategies, their single-case or small-series design precludes conclusions regarding reproducibility, comparative safety, or durability.

The five-year MITRAL trial results for balloon-expandable MViR and ViMAC showed sustained symptomatic improvement and stable gradients among survivors, but all-cause mortality exceeded 65% in both groups [4]. These findings reflect both the complexity of the intervention and the advanced comorbidity of treated patients. At present, Myval ViMAC should therefore remain confined to carefully selected, inoperable or extreme-risk patients treated at experienced centers with advanced CT planning, surgical backup, and access to LVOTO-prevention strategies.

### 4.5. Access, Deployment, and Adjunctive Techniques

Transseptal access is generally preferred because it avoids direct ventricular injury and has progressively replaced the transapical route. Transseptal puncture should be individualized according to left-atrial size, septal anatomy, delivery-system trajectory, and the intended landing zone; a posterior puncture with sufficient vertical height commonly facilitates coaxial advancement [8]. After septostomy, a pre-shaped stiff wire is positioned in the left ventricle while avoiding entrapment in the subvalvular apparatus.

Because the valve travels from the left atrium toward the ventricle, Myval crimping orientation must be the reverse of that used for retrograde aortic implantation. Deployment is performed under fluoroscopic and transoesophageal echocardiographic guidance, usually during rapid ventricular pacing. The target ventricular–atrial distribution of the frame depends on the surgical prosthesis or ring and the predicted neo-LVOT. Excessively ventricular implantation can increase LVOT encroachment, whereas an excessively atrial position may compromise anchoring and increase embolization risk.

Marked septal angulation or difficult alignment may be managed using supportive wires or, in selected cases, a through-and-through femoral venous–left ventricular apical rail. The latter preserves femoral valve delivery while using a limited apical access for wire externalization and trajectory control [18]. The available clinical experience does not validate a generation-specific advantage. In the three-patient hybrid cable-assisted series, Octapro was used in two mitral valve-in-valve procedures using 24.5- and 26 mm devices, whereas a 32 mm Octacor was used for valve-in-MAC [18]. One Octapro implantation achieved satisfactory positioning and hemodynamics; the other was complicated by initial malalignment and valve pop-out, requiring implantation of a second Octapro valve. Because valve generation co-varied with the anatomical substrate, only two patients received Octapro, the hybrid rail modified delivery mechanics, and no comparator or prospective measurement of intended versus achieved implantation depth was included, this experience supports feasibility but does not demonstrate a clinical benefit from reduced foreshortening. Transapical implantation remains an alternative when transseptal access is not feasible, but its invasiveness and association with less favorable outcomes in broader MViV experience support selective rather than routine use [40].

Preventive options for anticipated LVOTO include intentional anterior leaflet laceration using LAMPOON, balloon-assisted translocation of the mitral anterior leaflet, pre-emptive septal reduction, and selected leaflet-displacement techniques [42,43,44]. Myval-specific compatibility has been demonstrated both with tip-to-base LAMPOON in MViR [42] and with BATMAN in native ViMAC [43]. BATMAN involves electrosurgical perforation followed by balloon-mediated mechanical translocation of the anterior leaflet, thereby creating space for valve deployment while potentially contributing to prosthesis stabilization and sealing. However, compared with the controlled linear electrosurgical laceration produced by LAMPOON, mechanical leaflet tearing may be less predictable and could theoretically result in non-central disruption, tissue prolapse, inadequate sealing, or injury to the aortomitral curtain [43]. These complex adjunctive procedures should therefore remain confined to highly selected patients treated at centers with extensive TMVR experience.

### 4.6. Unresolved Issues and Evidence Gaps

The available evidence supports the technical feasibility of Myval MViV in selected high-risk patients, whereas evidence for MViR and particularly ViMAC remains preliminary. A systematic review reported a pooled technical success rate of approximately 96.9% for Myval mitral ViV/ViR procedures; however, this estimate was derived from small observational studies and case reports vulnerable to selection and publication bias [28].

Optimal antithrombotic therapy after mitral Myval implantation has not been established. The low-flow mitral environment, frequent atrial fibrillation, and large prosthetic frame may increase thrombosis susceptibility. Reported cases of gradient elevation or confirmed thrombosis responding to intensified anticoagulation support structured echocardiographic surveillance and prompt CT evaluation when leaflet thrombosis is suspected [13,15]. Treatment should nevertheless be individualized according to rhythm, bleeding risk, concomitant coronary indications, and existing prosthetic-valve recommendations.

Long-term structural durability, late frame interaction, reintervention feasibility, and the consequences of incomplete expansion remain unknown. The favorable results of contemporary Myval platforms in the aortic position cannot be extrapolated directly to mitral implantation because loading conditions, anchoring, frame deformation, and thrombogenicity differ [10,11]. Prospective multicenter registries should therefore report MViV, MViR, and ViMAC separately; incorporate independent CT and echocardiographic core laboratories; use standardized Mitral Valve Academic Research Consortium endpoints; and document valve size, expansion, implantation depth, access route, antithrombotic treatment, hemodynamics, thrombosis, reintervention, and long-term survival. Until such evidence becomes available, Myval use in the mitral position should be regarded as an anatomically selected application of a balloon-expandable aortic platform rather than an established dedicated transcatheter mitral replacement therapy. The principal Myval-specific mitral studies and their procedural, haemodynamic, and clinical outcomes are summarized in Table 3.

## 5. Tricuspid Applications

The use of Myval in the tricuspid position has been described predominantly for transcatheter tricuspid valve-in-valve (TViV) implantation within failed surgical bioprostheses and, less frequently, for valve-in-ring (TViR) treatment after failed annuloplasty. These applications should be distinguished from orthotopic transcatheter replacement of the native tricuspid valve using dedicated devices. The Myval frame requires a pre-existing prosthetic sewing ring, stent frame, or selected annuloplasty ring to provide anchoring and sealing; it is not designed for implantation within the large, non-calcified, and dynamically changing native tricuspid annulus.

### 5.1. Clinical Indications and Patient Selection

Patients presenting with structural deterioration of a tricuspid bioprosthesis are frequently younger than those treated for left-sided bioprosthetic failure and may have congenital heart disease, Ebstein anomaly, previous endocarditis, or multiple prior cardiac operations. Conversely, patients with failed tricuspid prostheses after left-sided valve surgery commonly have advanced right-sided heart failure, atrial fibrillation, pulmonary hypertension, hepatic or renal dysfunction, and a high predicted risk of surgical reintervention. In this context, TViV may be considered for symptomatic severe bioprosthetic stenosis, regurgitation, or mixed dysfunction when redo surgery is considered high risk by the Heart Team [2,7].

Assessment should extend beyond the severity of prosthetic dysfunction. Right-ventricular size and systolic function, pulmonary vascular disease, right atrial pressure, hepatic and renal reserve, nutritional status, frailty, and the likelihood of clinical recovery should be evaluated to avoid intervention at a stage of irreversible right-heart failure. Active infective endocarditis, intracardiac thrombus, uncontrolled infection, or absence of an adequate anchoring structure contraindicate implantation. Previous endocarditis that has been fully treated is not necessarily an absolute contraindication, although exclusion of active infection is mandatory.

Echocardiography defines the mechanism of failure and quantifies stenosis and regurgitation, while computed tomography identifies the surgical prosthesis or ring, measures its internal dimensions, and evaluates its relationship with the right coronary artery, right-ventricular free wall, venae cava, and pre-existing cardiac implantable electronic device leads. As in other valve-in-valve procedures, sizing should be based on the true internal diameter rather than the nominal surgical valve label [35]. The larger and intermediate Myval sizes may facilitate dimensional matching in large tricuspid prostheses, but this potential advantage has not been evaluated comparatively [29,32].

### 5.2. Tricuspid Valve-in-Valve Implantation

TViV is the most reproducible tricuspid application because the stented surgical bioprosthesis provides a circular and radiographically identifiable landing zone. The international tricuspid ViV registry, which principally included Melody and SAPIEN devices, demonstrated successful implantation in 150 of 152 attempted procedures, with 3.3% mortality at 30 days and substantial improvement in functional status [7]. These results support the general concept of transcatheter treatment for failed tricuspid bioprostheses but cannot establish the performance of Myval specifically.

Initial Myval experience consisted of individual reports. Karaduman et al. described transfemoral implantation of a 29 mm Myval within a failed 31 mm tricuspid bioprosthesis, with a mean gradient of 5 mmHg at discharge and 4 mmHg at one month [20]. Jensen et al. subsequently reported transjugular implantation of a 26 mm Myval within a degenerated 29 mm Mitroflow prosthesis [22]. The mean gradient decreased to 1.5 mmHg immediately after implantation and was 3 mmHg at one month, accompanied by improvement to New York Heart Association functional class I. These cases demonstrated the compatibility of Myval with both transfemoral and transjugular delivery but provided only short-term follow-up.

The largest dedicated Myval experience comprises five high-risk patients treated through transfemoral access [21]. The failed surgical prostheses had true internal diameters of 22–29 mm, and Myval sizes ranged from 24.5 to 29 mm, illustrating the use of intermediate sizing. Implantation was successful in all patients without post-dilatation or rapid pacing. Mean gradients decreased from 4–13 mmHg before intervention to 2–4 mmHg after implantation, and residual regurgitation was absent or trace. No stroke, myocardial infarction, permanent pacemaker implantation, or intraprocedural valve dysfunction was reported. One patient experienced femoral venous bleeding requiring transfusion and surgical treatment, and one patient died during the subsequent year. The remaining four patients showed symptomatic improvement without echocardiographic evidence of transcatheter valve deterioration at one year. Although informative, the small sample size, absence of independent event adjudication, and lack of a comparator prevent conclusions regarding relative safety or efficacy.

Myval TViV has also been reported in a 16-year-old patient with congenital heart disease and severe regurgitation of a 29 mm Hancock II prosthesis [45]. Following implantation of a 26 mm Myval, right atrial pressure decreased from 12–13 to 8–9 mmHg, with only trace residual regurgitation. Such use may defer further surgery in selected younger patients, but somatic growth, lifetime reintervention requirements, thrombosis, endocarditis, and long-term durability require particular consideration.

A systematic review identified six publications involving Myval in the tricuspid position and reported 100% procedural success [28]. This apparently favorable estimate should be interpreted cautiously because it was derived almost entirely from case reports and one five-patient series and is therefore highly susceptible to selection and publication bias.

### 5.3. Tricuspid Valve-in-Ring Implantation

TViR is substantially less predictable than TViV. Tricuspid annuloplasty rings are frequently incomplete, non-circular, rigid, or asymmetrical and may provide insufficient radial support along the septal portion of the annulus. Consequently, implantation can result in frame deformation, incomplete sealing, paravalvular regurgitation, ring disruption, or device embolization.

The first reported Myval TViR procedure involved implantation of a 32 mm valve within a partial annuloplasty ring [19]. Slow balloon expansion under rapid pacing was used to reduce ring deformation and displacement. The final mean gradient was 3.5 mmHg, with mild intravalvular and paravalvular regurgitation. A subsequent report described severe residual paravalvular regurgitation after implantation of a 26 mm Myval within an incomplete rigid ring, requiring closure with a dedicated paravalvular leak occluder [46]. Together, the cases show that valve deployment alone does not ensure circumferential sealing in incomplete rings. TViR should therefore be considered only after detailed CT assessment of ring type, internal area and perimeter, three-dimensional configuration, fluoroscopic visibility, and the location and dimensions of the uncovered annular segment. Balloon interrogation may provide additional information regarding ring expansion and sealing but cannot fully predict the behavior of an incomplete or rigid ring.

### 5.4. Procedural Technique

Access should be selected according to the orientation of the existing prosthesis, right atrial dimensions, caval anatomy, delivery-system characteristics, and operator experience. Transfemoral access has been used most frequently and may provide stable coaxial alignment through the inferior vena cava. However, severe right atrial enlargement and an acute inferior vena cava–tricuspid angle can complicate valve crossing. Transjugular access offers a shorter and potentially more direct trajectory in selected anatomies. Because Myval is crimped directly onto its delivery balloon outside the body, it may avoid some of the in-body valve-loading constraints encountered with other balloon-expandable systems during transjugular delivery [21,22].

The valve must be crimped in the appropriate orientation for antegrade blood flow from the right atrium to the right ventricle. A stiff guidewire is positioned across the surgical valve, commonly within a distal pulmonary artery branch, while avoiding right-ventricular free-wall injury. Fluoroscopic alignment uses the sewing ring or stent frame as the principal landmark and should be confirmed with transoesophageal or intracardiac echocardiography. In radiolucent prostheses, CT-derived fluoroscopic projections, multiple pigtail catheters, balloon markers, or fusion imaging may assist in defining the landing zone.

Rapid ventricular pacing is not invariably required because right-sided intracardiac pressures are lower than those encountered in aortic or mitral implantation. All five procedures in the dedicated Myval series were performed without pacing [21], whereas rapid pacing was used in other individual cases [19,22]. Deployment strategy should therefore be individualized according to valve stability, respiratory motion, and the risk of device displacement. Controlled inflation and temporary suspension of ventilation may improve positional accuracy.

Pre-existing transvenous leads require a predefined strategy. Implantation may entrap a lead between the transcatheter frame and surgical prosthesis, potentially causing lead dysfunction and making subsequent extraction difficult or impossible. Lead integrity, dependence on pacing, infection history, and alternative options—including lead extraction, coronary sinus pacing, epicardial systems, or leadless pacing—should be discussed before intervention.

### 5.5. Complications, Antithrombotic Therapy, and Evidence Gaps

Potential complications include malposition, embolization, frame underexpansion, residual stenosis, paravalvular regurgitation, right-ventricular perforation, vascular bleeding, lead entrapment, valve thrombosis, and infective endocarditis. In a broader 306-patient tricuspid ViV/ViR registry, the cumulative three-year incidences of death, reintervention, and valve-related adverse events were 17%, 12%, and 8%, respectively [47]. Eight cases each of endocarditis and clinically relevant valve thrombosis were reported. These data were obtained predominantly with other transcatheter valve platforms but identify complications that should also be considered during Myval follow-up.

No Myval-specific antithrombotic regimen has been established for the tricuspid position. The relatively low-flow right-sided circulation may increase thrombosis susceptibility, while many patients already have an indication for anticoagulation because of atrial arrhythmias or mechanical left-sided prostheses. Vitamin K antagonist therapy was used in the five-patient Myval series [21], but the optimal agent and duration remain uncertain. Treatment should therefore be individualized according to thrombotic and bleeding risks, with structured echocardiographic surveillance and CT assessment when an unexplained gradient increase or impaired leaflet motion is detected.

Current evidence supports the technical feasibility of Myval TViV in carefully selected patients, while evidence for TViR is limited to individual cases with variable sealing results. Dedicated prospective registries are required to define procedural success using standardized tricuspid endpoints, distinguish TViV from TViR, and report prosthesis type, true internal diameter, valve expansion, residual gradients, regurgitation, lead management, antithrombotic therapy, thrombosis, endocarditis, reintervention, and long-term durability. The available Myval-specific evidence in the tricuspid position is summarized in Table 4.

## 6. Pulmonary Applications

Transcatheter pulmonary valve implantation (TPVI) is an established alternative to repeat surgery for selected patients with right ventricular outflow tract (RVOT) dysfunction after congenital heart disease repair. Myval has been used in dysfunctional right ventricle-to-pulmonary artery conduits, failed surgical pulmonary bioprostheses, and selected native or surgically patched RVOTs. These anatomical substrates should be considered separately because a conduit or surgical valve provides a relatively defined landing zone, whereas a native or patched RVOT may be large, compliant, asymmetrical, and dynamically variable throughout the cardiac cycle.

### 6.1. Clinical Indications and Anatomical Selection

Most patients considered for Myval TPVI have undergone previous repair of tetralogy of Fallot, pulmonary atresia, truncus arteriosus, congenital pulmonary stenosis, a Ross or Rastelli procedure, or another operation involving RVOT reconstruction. Indications include severe pulmonary regurgitation, significant RVOT obstruction, or mixed dysfunction associated with symptoms, impaired exercise capacity, progressive right-ventricular dilatation or dysfunction, arrhythmias, or adverse hemodynamic consequences [1]. The decision to intervene should integrate symptoms, cardiopulmonary exercise testing, QRS duration and arrhythmia burden, right-ventricular pressure, and cardiac magnetic resonance measurements of pulmonary regurgitant fraction and right-ventricular volumes rather than relying on regurgitation severity alone.

Active infective endocarditis, intracardiac thrombus, an unsuitable or unstable landing zone, prohibitive coronary compression risk, or inability to protect a rupture-prone conduit generally preclude TPVI. In younger patients, body size, anticipated somatic growth, cumulative lifetime interventions, and the feasibility of future valve-in-valve procedures should also be considered.

Cardiac magnetic resonance remains the reference method for quantifying pulmonary regurgitation and right-ventricular volumes. Computed tomography complements magnetic resonance imaging by defining RVOT morphology, calcification, landing-zone dimensions, conduit integrity, branch pulmonary arteries, and the spatial relationship between the intended implant and the coronary arteries [37]. Static CT measurements alone are insufficient in compliant or patched RVOTs; dynamic balloon interrogation with simultaneous coronary angiography is frequently necessary to establish the stretched landing-zone diameter and exclude coronary compression.

### 6.2. Conduits and Surgical Pulmonary Bioprostheses

Dysfunctional conduits and stented pulmonary bioprostheses represent the most predictable pulmonary applications of Myval because their walls or prosthetic frames provide circumferential support. Valve selection should be based on the true internal diameter, residual lumen after degeneration, and the diameter achieved during balloon interrogation rather than the surgical label alone [35]. Calcification, previous stents, conduit angulation, and the proximity of the coronary arteries must also be assessed.

Initial evidence consisted of individual cases and small series demonstrating implantation within stenosed conduits, surgical bioprostheses, and selected native RVOTs [23,24,27]. In an early nine-patient series, Myval sizes of 23–29 mm were implanted following creation or confirmation of an appropriate landing zone, with no immediate significant valvular or paravalvular regurgitation and preserved valve function during a mean follow-up of approximately ten months [24].

The largest published multicenter Myval cohort included 53 patients with dysfunctional conduits, bioprosthetic pulmonary valves, or native/patched RVOTs [23]. Median age was 15 years, and prestenting was performed in 45 patients. The 29 and 32 mm valves were the most frequently used sizes, each accounting for approximately 40% of implantations. Procedural success was achieved in all patients, and the median peak instantaneous RVOT gradient decreased from 23.5 mmHg before implantation to 10 mmHg afterward. During a median follow-up of 360 days, three patients developed moderate pulmonary regurgitation. The observational design, heterogeneous RVOT substrates, limited follow-up, and absence of independent core-laboratory adjudication should be considered when interpreting these findings.

Evidence for the newer Myval Octacor configuration in the pulmonary position is more limited. A single-center series of ten patients with dysfunctional conduits or pulmonary bioprostheses reported a reduction in median right-ventricular systolic pressure from 68.5 to 33 mmHg and in peak RVOT gradient from 30 to 6.5 mmHg [26]. No frame fracture, conduit rupture, embolization, or endocarditis was observed during short-term follow-up. These findings establish technical feasibility but do not demonstrate comparative benefit or long-term durability.

### 6.3. Native and Patched Right Ventricular Outflow Tracts

Native or transannular-patch RVOTs present greater challenges because their geometry may be pyramidal, aneurysmal, tubular, or irregular, with marked variation between systole and diastole. Secure balloon-expandable valve implantation requires a reproducible constriction or a prestented cylindrical segment that can provide sufficient radial fixation. Maximum CT diameter alone may overestimate or underestimate the functional landing zone, particularly when different portions of the RVOT contain native muscle, surgical patch material, scar tissue, calcification, and compliant pulmonary arterial tissue.

The availability of 30.5 and 32 mm Myval valves has extended balloon-expandable TPVI to selected RVOTs beyond the conventional 29 mm limit. In a multicenter experience involving 17 patients with large native or reconstructed RVOTs, five received a 30.5 mm and 12 received a 32 mm Myval without prestenting [27]. Implantation was successful in all cases, with a mean postprocedural gradient of 11.4 ± 4.6 mmHg and no more than mild pulmonary regurgitation. Nevertheless, one patient developed pulmonary bleeding before implantation and subsequent stress cardiomyopathy, while another showed approximately 50% left anterior descending coronary artery compression after valve deployment despite remaining free of demonstrable ischemia. This event suggests that a negative balloon test may not exclude coronary interaction when the deployed valve exceeds the diameter reproduced during testing.

Custom-manufactured 35 mm Myval valves have been reported in five patients with extra-large native RVOTs. The first report described successful implantation with a dedicated 35 mm delivery balloon, with preserved valve competence and favorable right-ventricular remodeling at 11 months [50]. In a subsequent four-patient series, multimodality imaging and balloon interrogation identified functional landing zones of approximately 32–34.5 mm; prestenting was used in aneurysmal anatomies, whereas direct implantation was undertaken when a focal constriction provided a suitable landing zone [51]. Pulmonary regurgitation was eliminated with low residual gradients in all patients, although asymmetric expansion and significant residual regurgitation after the first implant necessitated deployment of a second 35 mm valve in one patient. These observations establish technical feasibility but not procedural safety, comparative effectiveness, or durability.

The custom-manufactured nominal 35 mm valve should be distinguished from use of the routinely available 32 mm Myval in extra-large RVOTs, including implantation within a landing zone prepared using a 35 mm balloon [52] and direct non-nominal expansion of the valve itself to approximately 35 mm [53]. Neither approach forms part of the routine 20–32 mm platform, and no Myval-specific bench evidence defines a safe overexpansion limit. Non-nominal expansion may produce asymmetric frame geometry, altered radial-force distribution, recoil, excessive foreshortening, skirt malapposition, instability, or fatigue-related frame fracture. Increased intercommissural spacing may also impair leaflet coaptation or mobility and increase tissue stress, potentially resulting in central regurgitation, leaflet injury, thrombosis, or accelerated structural deterioration. Accordingly, the custom 35 mm valve should be considered investigational and restricted to prospective studies or exceptional, jurisdictionally authorized compassionate use at experienced centers, with explicit informed consent and structured long-term surveillance.

### 6.4. Procedural Planning and Implantation Technique

Coronary compression represents the most consequential anatomical hazard of TPVI. CT should assess the proximity of the left anterior descending, left main, and right coronary arteries to the intended landing zone, including anomalous coronary courses frequently encountered in congenital heart disease. When any potential interaction exists, simultaneous selective coronary angiography during inflation of a non-compliant sizing balloon across the RVOT is required. Complete balloon occlusion also evaluates landing-zone stability, stretched diameter, residual contrast escape, and the potential for conduit or RVOT injury.

Prestenting is generally appropriate for severely calcified or irregular conduits, fracture-prone pre-existing stents, and native or patched RVOTs lacking a stable cylindrical segment. It can create a reproducible landing zone, reduce recoil, and protect the transcatheter frame from external compression. Covered stents should be immediately available when conduit rupture is possible. Conversely, additional prestenting may be unnecessary within some rigid surgical bioprostheses or in selected native RVOTs with a stable balloon-defined waist. The direct-implantation experience should not be generalized to compliant, aneurysmal, or poorly constrained anatomies [27].

Transfemoral venous access is most commonly used, although internal jugular access may be considered according to patient size and RVOT angulation. A stiff guidewire is positioned in a distal pulmonary artery branch, taking care to avoid distal vascular injury. The valve must be crimped in the correct orientation for antegrade flow, opposite to the orientation used for retrograde aortic implantation. A long delivery sheath may reduce interaction with the tricuspid valve, chordal apparatus, and right-ventricular free wall.

Deployment is performed under fluoroscopic guidance, frequently during rapid ventricular pacing or overdrive suppression of cardiac output. Controlled balloon inflation and temporary suspension of ventilation may improve positional stability. Immediately after implantation, angiography and hemodynamic assessment should document valve position, coronary patency, branch pulmonary artery flow, right-ventricular pressure, residual RVOT gradient, and pulmonary regurgitation. Echocardiography should additionally evaluate leaflet motion, paravalvular leakage, tricuspid valve injury, right-ventricular function, and pericardial effusion.

### 6.5. Complications, Durability, and Evidence Gaps

Potential complications include coronary compression, conduit rupture, pulmonary artery injury, device embolization, frame deformation, residual gradient or regurgitation, tricuspid apparatus injury, arrhythmias, vascular bleeding, valve thrombosis, and infective endocarditis. Prestent and valve-frame fractures should be assessed during longitudinal imaging, particularly in conduits exposed to external compression between the sternum and aorta.

No standardized Myval-specific antithrombotic regimen exists for the pulmonary position. Published series have used single or dual antiplatelet treatment with considerable variation in agent and duration. Therapy should be individualized according to thrombosis risk, bleeding risk, associated intracardiac prostheses, and concomitant indications for anticoagulation. Meticulous dental hygiene and infective endocarditis prophylaxis according to prosthetic-valve recommendations are essential.

The larger SAPIEN 3 pulmonary registry provides an important platform-level benchmark but cannot establish equivalence with Myval. Among 840 patients, implantation success was 98.5%; estimated six-year incidences of infective endocarditis, pulmonary valve replacement, and valve thrombosis were 3.8%, 8.0%, and approximately 0.7%, respectively [6]. Comparable long-term data are unavailable for Myval.

Current evidence supports the technical feasibility of Myval TPVI across several RVOT substrates, with the strongest data involving prestented conduits, surgical bioprostheses, and anatomically selected native RVOTs. Prospective studies should stratify outcomes by landing-zone type, valve generation and size, prestenting strategy, and degree of overexpansion; incorporate independent imaging assessment; and report coronary complications, frame integrity, endocarditis, thrombosis, right-ventricular reverse remodeling, exercise capacity, reintervention, and long-term structural valve durability. The available Myval-specific evidence in the pulmonary position is summarized in Table 5.

## 7. Post-Implantation Management

Post-implantation management after non-aortic Myval implantation should be individualized according to valve position, landing-zone characteristics, baseline rhythm, ventricular function, thromboembolic and bleeding risks, and the presence of concomitant prosthetic material or cardiac implantable electronic devices. Unlike conventional aortic transcatheter heart valve implantation, mitral, tricuspid, and pulmonary procedures involve markedly different loading conditions, flow velocities, anatomical constraints, and mechanisms of prosthetic dysfunction. Consequently, uniform extrapolation of aortic follow-up protocols or hemodynamic thresholds may be inappropriate. The principal objectives of post-procedural care are to establish a reliable hemodynamic baseline, prevent thrombotic and infective complications, identify early prosthetic dysfunction, and preserve the feasibility of future reintervention [2,28].

### 7.1. Early Post-Procedural Assessment

Comprehensive transthoracic echocardiography should be performed before discharge or within the early post-procedural period to document valve position, leaflet mobility, transprosthetic gradients, effective valve competence, and the presence and severity of paravalvular regurgitation. Ventricular function, pulmonary pressures, pericardial effusion, and interactions between the transcatheter prosthesis and adjacent structures should also be systematically evaluated. Transprosthetic gradients should be reported together with heart rate, rhythm, blood pressure, and, particularly for right-sided prostheses, loading conditions, because these variables substantially influence Doppler measurements.

Following mitral implantation, assessment should include the mean transmitral gradient, residual intravalvular or paravalvular regurgitation, left ventricular outflow tract gradient, left and right ventricular function, pulmonary artery pressure, and the hemodynamic relevance of any residual iatrogenic atrial septal defect. New hypotension, pulmonary oedema, hemolysis, or an unexpectedly elevated left ventricular outflow tract gradient should prompt immediate exclusion of prosthesis malposition, paravalvular leakage, or left ventricular outflow tract obstruction [13,15].

After tricuspid valve-in-valve or valve-in-ring implantation, the examination should focus on prosthetic leaflet motion, mean transtricuspid gradient, residual regurgitation, right ventricular size and systolic function, systemic venous congestion, and potential interaction with pre-existing transvenous pacing or defibrillator leads. Because transtricuspid gradients vary with respiration, heart rate, and volume status, serial comparison under comparable physiological conditions is essential [21,47]. Following pulmonary implantation, Doppler interrogation of the right ventricular outflow tract, quantification of pulmonary regurgitation, estimation of right ventricular pressure, and evaluation of right ventricular function should be complemented by assessment of branch pulmonary artery flow when technically feasible [26,27,50,51].

Clinical evaluation should include vascular or access-site complications, rhythm disturbances, hemoglobin and renal function, signs of hemolysis, congestion, and changes in functional status. These findings, together with the predischarge electrocardiographic and echocardiographic examinations, constitute the patient-specific reference against which subsequent changes should be interpreted.

#### Interpretation of Post-Implantation Hemodynamics

A low early post-implantation gradient supports satisfactory hemodynamic performance but cannot, in isolation, define procedural success or prosthetic dysfunction. Gradients must be interpreted against the predischarge baseline and in the context of valve size, flow, heart rate, rhythm, blood pressure, ventricular function, residual regurgitation, and—particularly on the right side—respiratory and loading conditions. A stable gradient may coexist with clinically important paravalvular regurgitation, malposition, leaflet thrombosis, or adjacent-structure obstruction; conversely, an isolated modestly elevated gradient may reflect high flow, tachycardia, patient–prosthesis mismatch, or incomplete frame expansion rather than structural valve dysfunction.

For mitral implantation, a mean transmitral gradient < 5 mmHg at a physiological heart rate is generally reassuring, whereas a persistent mean gradient ≥ 5 mmHg should prompt assessment for functional mitral stenosis, underexpansion, patient–prosthesis mismatch, leaflet thrombosis, or high-flow conditions. Gradients in the 5–10 mmHg range require contextual interpretation, particularly after implantation in small surgical bioprostheses or rings; a value ≥ 10 mmHg, or a meaningful increase from the early post-implantation baseline, is concerning and warrants comprehensive imaging. For tricuspid implantation, a mean transtricuspid gradient ≤ 5 mmHg is generally compatible with acceptable valve function, whereas a persistent gradient > 5 mmHg suggests possible tricuspid stenosis or prosthetic obstruction, provided that measurements are obtained under comparable respiratory, rhythm, and volume conditions. In pulmonary applications, an early mean RVOT gradient < 10 mmHg is usually reassuring; a gradient ≥ 20 mmHg, or an increase ≥ 10 mmHg from the established post-implantation baseline, should raise concern for residual or recurrent RVOT obstruction, frame underexpansion, stent recoil or fracture, thrombosis, or progressive conduit dysfunction.

Accordingly, reported gradients of approximately 2–4 mmHg after tricuspid valve-in-valve implantation and 4.6 ± 2.7 mmHg after mitral valve-in-valve implantation are compatible with favorable early hemodynamic performance. Pulmonary studies have also reported substantial gradient reductions, although mean and peak gradients are not interchangeable and should be interpreted at the individual-study level [16,21,23,25,26,27].

### 7.2. Antithrombotic Therapy

No randomized trial has evaluated antithrombotic therapy after Myval implantation outside the aortic position. The following strategy is therefore a pragmatic synthesis of contemporary prosthetic-valve guidance, reported non-aortic Myval practice, and position-specific thrombotic and bleeding considerations rather than a validated device-specific algorithm (Table 6).

The first decision is whether an independent indication for long-term oral anticoagulation exists. Patients with atrial fibrillation meeting contemporary stroke-risk criteria, venous thromboembolism, intracardiac thrombus, a mechanical prosthesis in another position, or another established indication should receive long-term oral anticoagulation irrespective of the Myval position. A vitamin K antagonist is required in patients with a mechanical prosthesis or clinically significant rheumatic mitral stenosis. In other eligible patients, either a vitamin K antagonist or a direct oral anticoagulant may be selected according to current guidance, renal and hepatic function, drug interactions, and bleeding risk [2]. Routine addition of antiplatelet therapy should be avoided unless required for a separate indication, such as recent acute coronary syndrome or percutaneous coronary intervention.

In the absence of an independent indication, oral anticoagulation with either a vitamin K antagonist or a direct oral anticoagulant is generally favored for at least 6 months after transcatheter mitral or tricuspid implantation when bleeding risk is acceptable [2]. For a vitamin K antagonist used solely for a bioprosthetic indication, a target international normalized ratio of 2.5 is generally applied. Continuation beyond 6 months may be considered after tricuspid implantation or when persistent thrombotic risk factors are present, including previous valve thrombosis, substantial prosthesis underexpansion, markedly impaired ventricular function, low-flow hemodynamics, or a recognized hypercoagulable state. Myval reports of elevated transmitral gradients or confirmed thrombosis responding to optimized anticoagulation and the use of vitamin K antagonists after tricuspid implantation remain hypothesis-generating and do not establish the superiority of a particular agent or duration [13,15,21].

High bleeding risk should prompt correction of modifiable factors, avoidance of unnecessary combination therapy, and closer clinical and laboratory surveillance. In patients without an independent indication for anticoagulation, a shortened approximately 3-month course after mitral or tricuspid implantation may be considered when the anticipated bleeding hazard outweighs the uncertain benefit of longer treatment. Early echocardiography, with gated computed tomography when leaflet thrombosis is suspected, should accompany any abbreviated regimen. If anticoagulation is contraindicated, single antiplatelet therapy or no antithrombotic treatment may be selected individually; neither approach has been established as an equivalent substitute for anticoagulation.

After pulmonary implantation, reported regimens have predominantly used aspirin alone or a limited course of dual antiplatelet therapy, with substantial variation between studies [23,24,25,26,27,50,51]. In patients without another indication for anticoagulation, single antiplatelet therapy for approximately 3–6 months is a reasonable pragmatic strategy, whereas routine dual antiplatelet therapy or oral anticoagulation cannot be supported solely on the basis of pulmonary Myval implantation. A shortened 1–3-month course, or no antithrombotic therapy when bleeding risk is prohibitive, may be considered after individualized assessment. Treatment should be reassessed at approximately 3 and 6 months according to rhythm, bleeding events, valve hemodynamics, leaflet motion, ventricular function, and any thromboembolic event.

### 7.3. Clinical and Imaging Surveillance

All patients with transcatheter bioprosthetic valves require lifelong clinical and echocardiographic surveillance. Current guidelines recommend transthoracic echocardiography within 3 months of implantation, at 1 year, and annually thereafter, with earlier reassessment whenever new symptoms, embolic events, hemolysis, infection, or suspected prosthetic dysfunction occur [2]. Given the off-label character and limited long-term evidence for non-aortic Myval applications, an additional evaluation at approximately 30 days and 6 months may be reasonable, particularly after mitral or tricuspid implantation, although this represents a pragmatic surveillance strategy rather than a Myval-specific evidence-based requirement.

Serial studies should be compared with the earliest high-quality post-implantation examination. Interpretation should focus on changes in gradients, leaflet mobility, regurgitation, ventricular function, pulmonary pressures, and clinical status rather than on isolated measurements. Transoesophageal echocardiography is indicated when transthoracic imaging is inconclusive or when thrombosis, endocarditis, paravalvular leakage, or prosthesis instability is suspected. Electrocardiographically gated cardiac computed tomography provides complementary information regarding hypoattenuated leaflet thickening, restricted leaflet motion, frame expansion, prosthesis position, and anatomical interaction with adjacent structures. Routine computed tomography screening for subclinical leaflet thrombosis is not currently recommended in asymptomatic patients with stable hemodynamics [2].

Pulmonary valve recipients, particularly those with congenital heart disease, may additionally require periodic cardiovascular magnetic resonance imaging to quantify pulmonary regurgitation, right ventricular volumes and function, and reverse remodeling [1]. Surveillance intervals should reflect the underlying congenital lesion, residual right ventricular outflow tract obstruction, ventricular dimensions, arrhythmia burden, and anticipated need for repeat intervention.

### 7.4. Evaluation and Management of Suspected Prosthetic Dysfunction

A new increase in transprosthetic gradient—particularly ≥5 mmHg in the mitral or tricuspid position, or ≥10 mmHg in the pulmonary position—new or worsening regurgitation, heart-failure symptoms, hemolysis, embolic events, or unexplained deterioration in ventricular function should initiate a structured evaluation. Potential mechanisms include leaflet thrombosis, infective endocarditis, structural valve deterioration, prosthesis underexpansion, malposition or migration, patient–prosthesis mismatch, and paravalvular leakage. Position-specific complications must also be considered, including left ventricular outflow tract obstruction after mitral implantation, interaction with pacing leads after tricuspid implantation, and recurrent right ventricular outflow tract obstruction or stent-related complications after pulmonary implantation.

Initial assessment should include clinical examination and comparison of current transthoracic echocardiography with the post-implantation baseline. Transoesophageal echocardiography and multiphase computed tomography should subsequently be selected according to the suspected mechanism. In hemodynamically stable patients with suspected infective endocarditis, multiple blood-culture sets should be obtained before antimicrobial treatment. Positron-emission tomography/computed tomography may provide additional diagnostic information when prosthetic valve endocarditis remains uncertain [2,54].

For clinically significant bioprosthetic valve thrombosis, therapeutic anticoagulation—generally with a vitamin K antagonist—is the preferred initial treatment in the absence of hemodynamic instability or another indication for immediate intervention [2]. Repeat imaging is required to document recovery of leaflet motion and normalization or improvement of valve hemodynamics. Indefinite anticoagulation may be considered following confirmed thrombosis when the recurrence risk outweighs bleeding risk. Persistent severe obstruction or regurgitation, prosthesis migration, uncontrolled infection, or progressive ventricular deterioration should prompt multidisciplinary evaluation by an experienced Heart Team. Depending on anatomy and mechanism, management options may include paravalvular leak closure, repeat transcatheter valve implantation, surgical intervention, or, in selected unstable patients, emergency treatment.

### 7.5. Endocarditis Prevention and Long-Term Considerations

Recipients of transcatheter prosthetic valves are considered at high risk for infective endocarditis. Meticulous oral and cutaneous hygiene, regular dental surveillance, and antibiotic prophylaxis before dental procedures involving manipulation of gingival or periapical tissues are therefore recommended [2,54]. Patients should be instructed to seek medical assessment for persistent fever, chills, unexplained malaise, or signs of systemic infection. Particular vigilance is warranted in tricuspid recipients with transvenous devices and in younger pulmonary valve recipients who may undergo repeated invasive procedures during lifelong congenital heart disease care.

Long-term durability of Myval prostheses in non-aortic positions remains undetermined. Follow-up should consequently capture structural valve deterioration, non-structural dysfunction, thrombosis, endocarditis, reintervention, heart-failure hospitalization, functional capacity, and survival. VARC-3 definitions provide a useful conceptual framework for distinguishing bioprosthetic valve dysfunction from bioprosthetic valve failure; however, these definitions were developed for aortic valve research and should not be transferred uncritically to the mitral, tricuspid, or pulmonary positions without anatomical and hemodynamic adaptation [55]. Prospective registries incorporating standardized imaging, position-specific hemodynamic criteria, antithrombotic exposure, and adjudicated clinical outcomes will be essential to define optimal surveillance and determine the long-term performance of Myval beyond the aortic position. Position-specific recommendations for post-implantation management and surveillance are summarized in Table 7.

## 8. Critical Appraisal and Research Agenda

The available evidence supports the technical feasibility of Myval implantation in selected non-aortic positions but does not establish comparative effectiveness, device-specific superiority, or long-term durability. Published experience remains dominated by case reports, small retrospective series, and a few observational cohorts from highly selected expert centers. Selection and publication bias, incomplete reporting of screened or excluded patients, and confounding by indication prevent procedural success among treated patients from being interpreted as evidence of broad anatomical applicability. Moreover, the theoretical value of intermediate sizing has not been shown to reduce underexpansion, residual gradients, paravalvular leakage, or embolization. Meaningful synthesis is further limited by the fundamentally different anchoring mechanisms and hemodynamics of mitral valve-in-valve, valve-in-ring, valve-in-MAC, tricuspid, and pulmonary interventions; inconsistent endpoint definitions; limited independent imaging or event adjudication; and follow-up that is insufficient to evaluate thrombosis, endocarditis, structural valve deterioration, and late reintervention. The marked imbalance in the volume of published Myval experience across non-aortic positions is shown in Supplementary Figure S1; study-level paired changes in reported transvalvular gradients are presented descriptively in Supplementary Figure S2.

### 8.1. Contextual Comparison with Established Transcatheter Platforms

No head-to-head study has compared Myval with SAPIEN or Melody in a non-aortic position. Cross-platform outcome data should therefore be interpreted as contextual benchmarks rather than estimates of relative treatment effect. The available cohorts differ substantially in anatomical substrate, patient age and operative risk, access route, screening and exclusion practices, device generation, procedural era, outcome definitions, duration of surveillance, and use of independent core laboratories or event adjudication. Most Myval reports additionally comprise small, highly selected series from experienced centers, whereas several SAPIEN and Melody benchmarks originate from large national or international registries. Accordingly, neither formal pooling nor unadjusted cross-study statistical testing would be methodologically appropriate.

In mitral valve-in-valve implantation, the 20-patient Myval series reported 100% technical success, a mean gradient of 6.3 ± 2.1 mmHg at 30 days, and 10% mortality at one year [16]. The corresponding SAPIEN 3 registry included 1529 patients and reported 96.8% technical success, a mean gradient of 7.0 ± 2.9 mmHg at one year, and mortality of 5.4% at 30 days and 16.7% at one year [40]. These results place the reported Myval hemodynamic and acute procedural outcomes within the numerical range observed with SAPIEN 3, but the approximately 75-fold difference in sample size, dissimilar case mix, and absence of adjusted comparison preclude an inference of equivalence. Dedicated Myval cohort-level outcomes are not available for mitral valve-in-ring or valve-in-mitral annular calcification implantation; comparison with the larger SAPIEN-family datasets is therefore not currently possible [4,41].

Similarly, all five patients in the Myval tricuspid valve-in-valve series underwent successful implantation, with post-procedural mean gradients of 2–4 mmHg, although one patient died during the subsequent year [21]. The international Melody/SAPIEN tricuspid valve-in-valve registry reported successful implantation in 150 of 152 attempted procedures and 3.3% mortality at 30 days [7]; in an expanded 306-patient cohort, the cumulative three-year incidences of death, reintervention, and valve-related adverse outcomes were 17%, 12%, and 8%, respectively [47]. In the pulmonary position, the largest multicenter Myval cohort included 53 patients with dysfunctional conduits, surgical pulmonary bioprostheses, or native or patched RVOTs [23]. Implantation was successful in all patients, and the median peak instantaneous RVOT gradient decreased from 23.5 mmHg (IQR, 10–53 mmHg) before implantation to 10 mmHg (IQR, 5–16 mmHg) after implantation. During a median follow-up of 360 days (IQR, 164–525 days), three patients developed moderate pulmonary regurgitation. By comparison, the international SAPIEN 3/Ultra registry included 840 patients and reported successful implantation in 827 patients (98.5%), a median post-procedural gradient of 6 mmHg (IQR, 2–12 mmHg), and absent or trivial pulmonary regurgitation in 96.4% [6]. Estimated six-year incidences of infective endocarditis, pulmonary valve replacement, and valve thrombosis were 3.8%, 8.0%, and 0.7%, respectively [6]. Although acute procedural and hemodynamic outcomes occupy a broadly similar numerical range, differences in RVOT substrate, prestenting, patient selection, endpoint definitions, sample size, and follow-up preclude conclusions regarding comparative safety, efficacy, or durability. These contextual, non-comparative cross-platform benchmarks are summarized in Table 8.

### 8.2. Future Research Directions

The most immediate requirement is prospective, multicenter evidence with a clearly defined denominator. International registries should use consecutive enrolment, maintain screening logs, document reasons for anatomical or clinical exclusion, and report mitral valve-in-valve, valve-in-ring, valve-in-MAC, tricuspid, and pulmonary applications separately. Where anatomically and clinically appropriate, Myval should be compared with established balloon-expandable platforms, Melody in the pulmonary position, and dedicated mitral or tricuspid replacement systems. Prospective concurrent controls would be preferable; when randomization is impractical, prespecified propensity-based analyses, target-trial emulation, or carefully constructed external controls should be used. Standardized computed-tomography and echocardiographic assessment, independent core laboratories, and blinded event adjudication are essential. Without these elements, procedural success among selected recipients cannot establish anatomical eligibility, comparative effectiveness, or device-specific superiority.

A parallel requirement is rigorous evaluation of sizing and device–landing-zone interaction. Integrated bench testing, computational modeling, and prospective procedural imaging should examine nominal diameter, true internal diameter, oversizing, frame underexpansion and eccentricity, radial-force distribution, anchoring, sealing, leaflet kinematics, skirt apposition, and final axial frame position. Planned and achieved implantation depth should be recorded in millimeters. Intermediate sizes should be compared with the nearest conventional sizes, while Octapro should be evaluated directly against Octacor and first-generation Myval. Dedicated evaluation is particularly necessary for the custom-manufactured nominal 35-mm valve and for non-nominal expansion of the routinely available 32-mm valve to approximately 35 mm, which represent distinct device configurations and should not be considered interchangeable [50,51,53]. Bench studies should define their final frame geometry, radial strength, recoil, foreshortening, fatigue resistance, skirt apposition, leaflet coaptation and mobility, hydrodynamic performance, and accelerated wear. Prospective clinical studies should then determine whether the expanded size matrix or modified frame architectures reduce migration or embolization, paravalvular regurgitation, residual gradients, left ventricular outflow tract obstruction, annular or conduit injury, coronary or aortic compression, and the need for a second valve. Until such evidence is available, neither the custom 35-mm valve nor overexpansion beyond a valve’s nominal diameter should be regarded as a routine treatment strategy.

Long-term investigation should focus on thrombosis, antithrombotic therapy, structural valve deterioration, infective endocarditis, and reintervention. Mitral and tricuspid studies should document anticoagulant selection and duration and should incorporate serial echocardiography and gated computed tomography to evaluate clinical valve thrombosis, hypoattenuated leaflet thickening, restricted leaflet motion, thromboembolic events, and bleeding. Pulmonary studies should additionally assess frame fracture, right-ventricular remodeling, exercise capacity, endocarditis, and freedom from reintervention. Follow-up should extend for at least 5 years in mitral and tricuspid cohorts and preferably 10 years in younger pulmonary populations because durability cannot be inferred from stable early gradients or short-term freedom from reintervention [1,26,27,50,51]. Subsequent valve-in-valve feasibility and preservation of future treatment options should also be evaluated as components of lifetime valve management.

Outcome reporting should use position-appropriate adaptations of VARC-3, TVARC, and MVARC definitions, while a dedicated pulmonary endpoint framework remains necessary [55,57,58]. Until comparative effectiveness, anatomy-specific sizing, and long-term valve safety have been established, Myval should be regarded as a technically versatile option for carefully selected patients rather than a preferred platform for non-aortic implantation.

## 9. Conclusions

Myval implantation in mitral, tricuspid, and pulmonary positions is feasible in selected patients, but the evidence remains predominantly observational and is insufficient to establish comparative superiority or long-term durability. The platform’s nine-size matrix may facilitate prosthesis–landing-zone matching, particularly within circular surgical bioprostheses and prepared right ventricular outflow tracts, whereas outcomes are less predictable in non-circular annuloplasty rings, severe mitral annular calcification, and native or patched outflow tracts. Procedural success depends on multimodality imaging, substrate-specific sizing, secure anchoring and sealing, protection of adjacent structures, and position-specific follow-up. These procedures are generally off-label, with regulatory status varying by jurisdiction, and should be considered on an individual basis by experienced multidisciplinary Heart Teams. Prospective multicenter studies using standardized imaging, independent outcome adjudication, appropriate comparators, and long-term surveillance are required to define safety, thrombosis, durability, and the role of Myval in lifetime valve management.

## Figures and Tables

**Table 1:** Myval platform generations and design attributes relevant to non-aortic implantation.

Platform	Leaflets and Frame	Frame Geometry and Sealing	Nominal Size Matrix	Delivery System	Potential Non-Aortic Relevance and Limitations
First-generation Myval	Three decellularized bovine-pericardial leaflets; radiopaque MP35N nickel–cobalt frame; intra-annular leaflet position.	Hybrid honeycomb frame: relatively open outflow cells and denser closed inflow cells. Internal PET cuff plus external PET skirt at inflow. Reported deployment foreshortening approximately 21–24%.	Routine matrix: 20, 21.5, 23, 24.5, 26, 27.5, 29, 30.5, and 32 mm.	Navigator balloon catheter; direct crimping; dog-bone inflation profile; 14-Fr expandable Python sheath for the contemporary 20–32 mm platform.	The nine nominal diameters provide finer sizing granularity and may reduce the numerical discrepancy between the selected valve and measured landing zone; no non-aortic study has demonstrated fewer embolizations, paravalvular leaks, residual gradients, or reinterventions. Balloon expansion provides immediate radial fixation but no recapture after inflation starts.
Myval Octacor	Second-generation balloon-expandable platform retaining bovine-pericardial leaflets, metal frame, and the nine-size 20–32 mm matrix.	Two rows of interlacing octagonal cells; enlarged internal PET cuff and broader external skirt; landing-zone marker. Reported foreshortening approximately 19–20%.	20–32 mm, including intermediate and extra-large sizes.	Navigator Inception delivery system; 14-Fr expandable Python sheath.	Lower foreshortening and a marker may improve depth predictability; the larger skirt may improve sealing of small channels but cannot compensate for major ring discontinuity or dehiscence.
Myval Octapro	Third-generation cobalt-alloy platform; intra-annular trileaflet bovine-pericardial valve; total frame height reported as 19.5–23.5 mm across sizes.	Two-row hybrid design: V-shaped chevrons in the outflow zone (55% of frame height) and interlacing octagons at inflow; internal PET skirt and higher external skirt. Reported foreshortening approximately 10–12%.	20–32 mm, same nine nominal diameters.	Navigator Pro delivery system; 14-Fr expandable Python sheath.	Reduced foreshortening is mechanistically relevant to short landing zones and planned implantation depth. Non-aortic clinical evidence is currently limited to case-level mitral experience.
Custom 35 mm Myval	Custom-manufactured balloon-expandable Myval derivative; not part of the routine 20–32 mm commercial matrix.	Intended for nominal 35 mm deployment. Separately, off-label expansion of a routine 32 mm Myval to approximately 35 mm has been reported; expansion beyond 35 mm has not been established.	35 mm nominal; custom/investigational availability and regulatory status are jurisdiction-specific.	Navigator-based delivery has been reported; procedural equipment and inflation volume are individualized.	May extend therapy to extra-large native RVOTs, but asymmetric expansion, residual regurgitation, and need for a second valve have occurred. Do not generalize overexpansion thresholds.

Abbreviations: PET, polyethylene terephthalate; RVOT, right ventricular outflow tract.

**Table 3:** Key published Myval-specific clinical evidence in the mitral position.

Study and Design	Substrate and Sample	Access and Device Strategy	Technical and Hemodynamic Findings	Clinical Outcomes and Follow-Up
Ielasi et al., 2021 [14] Case series	Mixed aortic/mitral ViV, *n* = 5 total; mitral subset not separately extractable from the abstract.	Balloon-expandable Myval; access and size varied by case.	Demonstrated early feasibility of Myval ViV in more than one left-sided position.	Early procedural follow-up only; mitral-specific outcomes not separable.
Blasco-Turrión et al., 2022 [13] Multicenter retrospective series	MViV, N = 11.	Transfemoral–transseptal; Myval 23–29 mm; intermediate sizes used in 54%.	Author-defined technical success 100%. Mean mitral gradient 14.7 ± 2.3 to 3.4 ± 1.7 mmHg; no LVOT obstruction, migration, or PVL reported.	One major vascular complication. At 6 months, one gradient rose to 15 mmHg during suboptimal anticoagulation and improved after optimization.
Moscarella et al., 2023 [15] Multicenter retrospective cohort	Left-sided ViV/ViR, N = 97 total; mitral *n* = 64; MViV and MViR not fully separated.	Myval across multiple centers; transseptal mitral implantation predominated.	Overall technical success 98%. Two acute mitral THV dysfunctions required a second valve.	Mitral survival approximately 89% at median 15 months (IQR 8–21). Delayed severe LVOT obstruction and valve thrombosis were reported; thrombosis responded to warfarin.
Kılıç et al., 2021 [42] Case report	MViR, N = 1; high predicted LVOT-obstruction risk.	Transseptal 26 mm Myval with tip-to-base LAMPOON; predicted neo-LVOT 169 mm^2^.	Successful implantation without LVOT obstruction.	Posterior PVL and iatrogenic atrial septal defect required percutaneous closure 1 week later; clinical improvement at discharge.
Sankardas et al., 2024 [16] Single-center retrospective series	MViV, N = 20.	Transcatheter Myval MViV; device sizes individualized to the failed prosthesis.	Technical success 100%; mean gradient 4.6 ± 2.7 mmHg after implantation and 6.3 ± 2.1 mmHg at 30 days; no significant PVL or LVOT obstruction.	One-year mortality 10%; survivors were predominantly NYHA class I–II.
Ninios et al., 2025 [43] Case report	ViMAC, N = 1; severe circumferential MAC (score 9), AML 28 mm, predicted neo-LVOT 80 mm^2^.	Transfemoral–transseptal 29 mm Myval; BATMAN leaflet translocation.	No transmitral gradient, systolic LVOT gradient, or PVL after implantation despite minor ventricular-frame protrusion.	Immediate procedural result only.
Kılıç et al., 2026 [17] Case report	ViMAC, N = 1; prohibitive surgical risk.	Transseptal 30.5 mm Myval.	Successful anchoring with no reported PVL or LVOT obstruction.	Stable early follow-up; longer-term durability not established.
Nasso et al., 2026 [18] Three-case technical series	Hybrid cable-assisted TMVI, N = 3: ViMAC n = 1 and degenerated surgical bioprostheses n = 2.	Myval Octacor 32 mm; Myval Octapro 24.5 and 26 mm; hybrid cable used to improve support/coaxiality.	All met author-defined technical success; gradients 4.5–5.8 mmHg; no significant PVL, LVOT obstruction, embolization, or stroke.	One initial malalignment/early migration required a second valve; survivors discharged NYHA I–II.

Abbreviations: AML, anterior mitral leaflet; BATMAN, balloon-assisted translocation of the mitral anterior leaflet; IQR, interquartile range; LAMPOON, laceration of the anterior mitral leaflet to prevent outflow obstruction; LVOT, left ventricular outflow tract; MAC, mitral annular calcification; MViR, mitral valve-in-ring; MViV, mitral valve-in-valve; NYHA, New York Heart Association; PVL, paravalvular leak; THV, transcatheter heart valve; TMVI, transcatheter mitral valve implantation; ViMAC, valve-in-MAC.

**Table 4:** Key published Myval-specific clinical evidence in the tricuspid position.

Study and Design	Substrate and Sample	Access and Device Strategy	Technical and Hemodynamic Findings	Clinical Outcomes and Follow-Up
Karaduman et al., 2021 [20] Case report	TViV, N = 1; failed 31 mm surgical tricuspid bioprosthesis.	Transfemoral 29 mm Myval.	Successful implantation; mean tricuspid gradient 5 mmHg at discharge and 4 mmHg at 1 month.	Early symptomatic/hemodynamic success; no long-term data.
Ayhan et al., 2022 [19] Case report	TViR, N = 1; failed partial/incomplete tricuspid ring.	Transfemoral 32 mm Myval; slow deployment under rapid pacing.	Final mean gradient 3.5 mmHg; mild residual intravalvular/paravalvular regurgitation reported.	Short case-level follow-up.
Jensen et al., 2022 [22] Two-case report; one Myval case	TViV; Myval case n = 1 in a failed 29 mm Mitroflow prosthesis.	Transjugular 26 mm Myval; rapid pacing at 180 bpm.	Mean gradient 11 to 1.5 mmHg immediately and 3 mmHg at 1 month.	NYHA III to I at 1 month. A transvenous lead was ultimately passed across the new valve without reported regurgitation.
Mussayev et al., 2023 [21] Single-center case series	TViV, N = 5; surgical true internal diameters 22–29 mm.	All transfemoral; Myval 24.5–29 mm; no rapid pacing or post-dilatation.	Post-implant mean gradients 2–4 mmHg; none/trace TR.	One access-site bleed required transfusion and surgery. One death by 1 year, cause not reported; the other four had favorable clinical status. VKA used in all.
Assaad et al., 2024 [46] Case report	TViR, N = 1; incomplete rigid 26 mm Contour 3D ring.	26 mm Myval; staged percutaneous closure of residual PVL.	Severe PVL through the open septal segment (defect 20 × 14 mm) required an 18 × 10 mm plug 2 weeks later.	Trivial/trace residual leak and symptomatic improvement at 6 months; RV dysfunction persisted.
Öztürk et al., 2025 [45] Pediatric case report	TViV, N = 1; 16-year-old with failed 29 mm Hancock II prosthesis.	Balloon interrogation/stabilization (25 × 40 mm); 26 mm Myval.	Mean RA pressure 12–13 to 8–9 mmHg; trace TR.	Discharged after rhythm management with electrophysiologic overdrive pacing; no long-term durability data.
Sadikoglu et al., 2025 [48] Case report	TViR, N = 1; failed annuloplasty ring.	30.5 mm Myval with 2-mL overfilling; post-dilatation for PVL; stiff wire positioned in left PA.	Final valve position and sealing were satisfactory after post-dilatation.	Stiff-wire PA perforation caused massive hemoptysis and hemodynamic collapse; rescued with balloon tamponade and Amplatzer Vascular Plug 4. Stable at 6 months.
Farandzha et al., 2025 [49] Three-case series	TViV, N = 3; fluoroscopically invisible surgical tricuspid prostheses.	Myval 27.5, 32, and 30.5 mm; RCA guidewire landmark plus balloon-waist localization.	All three implants successful; mean gradients 4–5 mmHg; no residual TR.	One sinoatrial block required an atrial pacemaker; follow-up was short and not standardized.

Abbreviations: NYHA, New York Heart Association; PA, pulmonary artery; PVL, paravalvular leak; RA, right atrium; RCA, right coronary artery; RV, right ventricle; TR, tricuspid regurgitation; TViR, tricuspid valve-in-ring; TViV, tricuspid valve-in-valve; VKA, vitamin K antagonist.

**Table 5:** Key published Myval-specific clinical evidence in the pulmonary position.

Study and Design	RVOT Substrate and Sample	Landing-Zone and Device Strategy	Technical and Hemodynamic Findings	Clinical Outcomes and Follow-Up
Sivaprakasam et al., 2021 [25] Multicenter series	Prestented stenotic RV–PA conduits, N = 7.	23 mm Myval in all; one second 24.5 mm valve for residual PR.	Conduit diameter 9.3 ± 2.8 to 20.8 ± 1.1 mm after prestenting; gradient 87.3 ± 31.7 to 12.7 ± 6.4 mmHg after prestenting/implantation.	Median follow-up 16 months (range 10–22): all asymptomatic, no PR, gradient 12.5 ± 5.8 mmHg. DAPT reported.
Odemis and Yenidogan, 2022 [24] Single-center series	N = 9: conduits n = 3; native RVOT n = 6.	All prestented (same session n = 2; staged in others); valves 23 mm in conduits, 26 mm n = 4 and 29 mm n = 2 in native RVOTs.	All implants successful; no immediate leak, dysrhythmia, or frame fracture.	Mean follow-up 9.8 months (range 6–14); competent valves and RV–PA gradient < 15 mmHg in all. Aspirin for 1 year reported.
Rodríguez Ogando et al., 2022 [52] Case report	Large native RVOT after tetralogy-of-Fallot repair, N = 1.	RVOT prestented with an XXL stent on a 35 mm balloon; 32 mm Myval.	Successful implantation with no in-hospital complication.	At 6 months, good valve function without PR or PVL.
Houeijeh et al., 2023 [53] Case report	Large, tortuous native RVOT (approximately 35 mm) with severe biventricular dysfunction, N = 1.	Complex prestenting; 32 mm Myval expanded to approximately 35 mm under local anesthesia; 3D modeling aided planning.	Successful implantation with satisfactory immediate function.	Short follow-up; long-term effects of overexpansion unknown.
Al Nasef et al., 2024 [23] Multicenter observational cohort	N = 53: stenotic conduits, transannular-patch RVOTs, and surgical pulmonary bioprostheses.	Prestenting n = 45; prior surgical valve n = 6; 29 and 32 mm valves each n = 22 (40%).	Procedural success 100%; median peak instantaneous gradient 23.5 (IQR 10–53) to 10 mmHg (IQR 5–16).	Median follow-up 360 days (IQR 164–525); three patients had moderate neo-PR; no more-than-mild TR at last follow-up.
Ramakrishnan et al., 2024 [50] Case report	Extra-large native RVOT after repaired tetralogy of Fallot, N = 1.	Custom-made 35 mm Myval.	Successful implantation with elimination of significant PR.	Short case-level follow-up.
Manica et al., 2025 [27] Multicenter retrospective series	Large native/reconstructed RVOT, N = 17; no prestenting.	Direct implantation: 30.5 mm n = 5; 32 mm n = 12.	Procedural success 100%; post-implant mean RVOT gradient 11.4 ± 4.6 mmHg; mild PR n = 1.	One pulmonary bleed occurred before implantation with subsequent Takotsubo syndrome; one approximately 50% LAD compression without ischemia. Immediate results only.
Badawy et al., 2025 [26] Single-center retrospective series	Dysfunctional RV–PA conduits or pulmonary bioprostheses, N = 10.	Myval Octacor; size individualized to the landing zone.	Median RV systolic pressure 68.5 to 33 mmHg; peak gradient 30 to 6.5 mmHg.	No reported frame fracture, conduit rupture, embolization, or endocarditis during short follow-up.
Al Farqani et al., 2026 [51] Four-case series	Extra-large native RVOTs with severe PR, N = 4.	Custom 35 mm Myval; balloon interrogation identified a functional landing zone around 32 mm; prestenting selected by anatomy.	Procedural success in all; peak gradients approximately 4–7 mmHg. One asymmetric first implant with residual PR required a second 35 mm valve.	Early clinical outcomes only; no major periprocedural complication reported.

Abbreviations: DAPT, dual antiplatelet therapy; IQR, interquartile range; LAD, left anterior descending coronary artery; PA, pulmonary artery; PR, pulmonary regurgitation; PVL, paravalvular leak; RV, right ventricle; RVOT, right ventricular outflow tract; TR, tricuspid regurgitation.

**Table 6:** Proposed position-, rhythm-, and bleeding-risk-stratified antithrombotic algorithm after non-aortic Myval implantation.

Clinical Branch	Mitral: MViV, MViR or ViMAC	Tricuspid: TViV or TViR	Pulmonary: Conduit, Bioprosthesis or RVOT	Reassessment and Qualification
AF with an indication for OAC, or another long-term OAC indication; acceptable bleeding risk	Long-term OAC alone	Long-term OAC alone	Long-term OAC alone	Do not add antiplatelet therapy unless separately indicated. VKA is mandatory for a mechanical prosthesis or clinically significant rheumatic mitral stenosis.
AF/other OAC indication with high bleeding risk	OAC generally remains indicated; correct modifiable bleeding factors and intensify monitoring	Same	Same	Bleeding risk alone should not automatically eliminate stroke-prevention therapy. Temporarily interrupt OAC only for active or clinically significant bleeding.
No independent OAC indication; acceptable bleeding risk	OAC for at least 6 months; discontinue thereafter if valve function is stable and no new indication has developed	OAC for at least 6 months; consider extended or indefinite treatment when thrombotic risk remains high and bleeding risk is low	SAPT, generally aspirin, for approximately 3–6 months	Reassess rhythm, gradients, regurgitation, leaflet motion and bleeding at approximately 3 and 6 months.
No independent OAC indication; high bleeding risk	Consider a shortened approximately 3-month OAC course with early imaging; if OAC is contraindicated, individualized SAPT or no therapy	Same; prolonged OAC is generally avoided unless thrombotic risk is compelling	SAPT for approximately 1–3 months; no therapy may be considered when bleeding risk is prohibitive	Abbreviated or absent therapy is an expert compromise, not an evidence-based equivalent strategy.
Confirmed or strongly suspected bioprosthetic valve thrombosis	Therapeutic anticoagulation, generally with a VKA, and repeat imaging	Same	Same	Repeat TTE and/or gated CT after approximately 6–12 weeks. Consider prolonged or indefinite OAC after recurrent thrombosis if bleeding risk permits.

Abbreviations: AF, atrial fibrillation; CT, computed tomography; MViR, mitral valve-in-ring; MViV, mitral valve-in-valve; OAC, oral anticoagulation; RVOT, right ventricular outflow tract; SAPT, single antiplatelet therapy; TTE, transthoracic echocardiography; TViR, tricuspid valve-in-ring; TViV, tricuspid valve-in-valve; ViMAC, valve-in-mitral annular calcification; VKA, vitamin K antagonist.

**Table 7:** Position-specific post-implantation management and surveillance after non-aortic Myval implantation.

Position	Predischarge Reference Dataset	Antithrombotic Strategy	Routine Imaging Surveillance	Red Flags and Escalation	Position-Specific Priorities
All positions	Clinical examination, ECG, blood count, renal function, and predischarge TTE establish the reference state. Record valve size/generation, access route, implant depth, gradients, regurgitation, ventricular function, and complications.	No Myval-specific randomized antithrombotic regimen exists. Base therapy on valve position, atrial fibrillation/other OAC indications, bleeding risk, recent PCI, and local prosthetic-valve guidance.	TTE before discharge; approximately 30 days; 3–6 months; 12 months; then annually. Earlier imaging if symptoms or hemodynamics change. Compare every study with the first stable baseline.	New or increasing gradient/regurgitation, new HF, hemolysis, fever/bacteremia, embolic event, or suspected migration → prompt TTE plus TEE and/or gated CT according to the question.	Meticulous dental hygiene; infective-endocarditis prevention for prosthetic-valve patients according to contemporary guidance [54]. Provide the patient with device details and an emergency contact plan.
Mitral MViV, MViR, ViMAC	Document transmitral mean gradient at heart rate, residual MR/PVL, LVOT gradient, iatrogenic atrial septal defect/shunt, pulmonary pressure, and LV/RV function.	OAC is commonly favored for at least the early post-TMVR period and continued when another indication exists; exact agent/duration remain untested for Myval. Antiplatelet addition should require a separate indication because bleeding risk is substantial.	Use TEE or gated CT when gradients rise, leaflet motion is uncertain, PVL is clinically important, or thrombus/HALT is suspected. Reassess iatrogenic septal shunt and pulmonary pressure.	Thrombosis or HALT; delayed LVOT obstruction; PVL/hemolysis; migration; high residual gradient; endocarditis. Suspected thrombosis requires imaging confirmation and individualized anticoagulation rather than empiric permanent therapy.	MViR and ViMAC need a lower threshold for early reassessment because geometry, sealing, and anchoring are less predictable than MViV.
Tricuspid TViV or TViR	Record mean gradient with heart rate and respiratory phase, residual TR/PVL, RA/RV size and function, venous/hepatic congestion, rhythm, and interaction with any transvenous lead.	Right-sided low-flow conditions and atrial arrhythmia may increase thrombosis risk; VKA was used in the largest small Myval series [21], but no comparative regimen exists. Individualize OAC and avoid unsupported combination therapy.	TTE at the standard intervals; TEE/CT if acoustic windows are inadequate or thrombosis/PVL is suspected. Reassess pacing strategy before any new lead crosses the valve.	Increasing gradient, recurrent TR/PVL, right HF, lead interference, thrombosis, migration, conduction disturbance, access-site complication, or endocarditis.	TViR—especially incomplete rings—requires targeted PVL surveillance. Persistent RV dysfunction may limit symptomatic recovery despite technically successful valve implantation.
Pulmonary Conduit, bioprosthesis, or native/patched RVOT	Record peak and mean RVOT gradients, PR/PVL, RV pressure, RV size/function, branch-PA flow, rhythm, and any prestent/frame abnormality. Establish congenital-heart-disease imaging baseline.	Published Myval series used heterogeneous SAPT or DAPT regimens [23,24,25,26,27,51]; there is no evidence for routine OAC solely because a Myval valve is in the pulmonary position. Tailor therapy to concomitant indications and bleeding risk.	Lifelong congenital-heart-disease follow-up. TTE at early and annual visits; CMR commonly at approximately 6–12 months and periodically thereafter for RV volumes/function and PR; CT/fluoroscopy if frame integrity, coronary interaction, or landing-zone change is suspected.	Rising gradient, recurrent PR, frame fracture, stent/conduit recoil, coronary symptoms, arrhythmia, RV deterioration, endocarditis, or need for reintervention.	Younger age makes cumulative endocarditis, frame integrity, leaflet durability, coronary access, and future valve-in-valve strategy central to surveillance.

Abbreviations: CMR, cardiovascular magnetic resonance; CT, computed tomography; DAPT, dual antiplatelet therapy; ECG, electrocardiography; HALT, hypoattenuated leaflet thickening; HF, heart failure; LV, left ventricle; LVOT, left ventricular outflow tract; MR, mitral regurgitation; MViR, mitral valve-in-ring; MViV, mitral valve-in-valve; OAC, oral anticoagulation; PA, pulmonary artery; PCI, percutaneous coronary intervention; PR, pulmonary regurgitation; PVL, paravalvular leak; RA, right atrium; RV, right ventricle; RVOT, right ventricular outflow tract; SAPT, single antiplatelet therapy; TEE, transoesophageal echocardiography; TMVR, transcatheter mitral valve replacement; TR, tricuspid regurgitation; TTE, transthoracic echocardiography; TViR, tricuspid valve-in-ring; TViV, tricuspid valve-in-valve; ViMAC, valve-in-MAC; VKA, vitamin K antagonist.

**Table 8:** Contextual outcome benchmarks for Myval and established transcatheter platforms in corresponding non-aortic landing zones.

Application	Platform and Study	Population and Follow-Up	Procedural Outcome	Hemodynamic or Valve Outcome	Clinical Outcome and Interpretation
Mitral valve-in-valve	Myval; [16]	20 treated; follow-up to 1 year	Technical success 20/20 (100%)	Mean gradient 4.6 ± 2.7 mmHg post-implantation and 6.3 ± 2.1 mmHg at 30 days; no significant valvular regurgitation, PVL, or LVOTO	One-year mortality 2/20 (10%); all survivors in NYHA class I or II. Small, retrospective, single-center series without a comparator
Mitral valve-in-valve	SAPIEN 3; [40]	1529 treated at 295 centers; 1-year analysis	Technical success 1480/1529 (96.8%)	Mean gradient 7.0 ± 2.9 mmHg at 1 year	Mortality 5.4% at 30 days and 16.7% at 1 year. Large registry benchmark, but case mix and outcome timing differ from the Myval series
Mitral valve-in-ring	Myval	No dedicated cohort with separately reported outcomes	Not estimable	Not estimable	Existing Myval evidence is limited to individual reports or mixed cohorts; quantitative comparison is not possible
Mitral valve-in-ring	SAPIEN-family balloon-expandable THVs; [41]	820 treated; 1-year analysis	Technical success 88.0%	Mean gradient 8.4 ± 3.4 mmHg; ≤mild residual MR in 91.7% at 1 year	Mortality 8.3% at 30 days and 22.4% at 1 year; mitral reintervention 9.1% at 1 year
Valve-in-MAC	Myval	Individual cases only	No cohort-level estimate	No cohort-level estimate	Case-level feasibility cannot define technical success, LVOTO, embolization, mortality, or durability rates
Valve-in-MAC	SAPIEN-family balloon-expandable THVs; MITRAL trial [4]	31 prospectively treated; follow-up to 5 years	Technical success 74.2%	Mean gradient 6.7 ± 2.5 mmHg among survivors at 5 years	Mortality 16.7% at 30 days, 34.5% at 1 year, and 67.9% at 5 years; reflects the high-risk substrate rather than device performance alone
Tricuspid valve-in-valve	Myval; [21]	5 treated; follow-up to 1 year	Successful implantation in 5/5	Post-implantation mean gradients 2–4 mmHg; residual TR absent or trace	One death during the subsequent year; four patients improved without reported THV deterioration. Cause of death was not specified
Tricuspid valve-in-valve	Melody/SAPIEN; [7,47]	152 attempts in initial registry; 306 patients in expanded cohort; median expanded-cohort follow-up 15.9 months	Successful implantation in 150/152 attempts (98.7%)	Significant improvement in tricuspid inflow gradient and regurgitation	Thirty-day mortality 3.3%; cumulative 3-year death 17%, reintervention 12%, and valve-related adverse outcome 8%. Mixed Melody/SAPIEN and valve-in-valve/valve-in-ring dataset
Pulmonary; mixed RVOT substrates	Myval; [23]	53 patients; conduits, surgical pulmonary bioprostheses, and native or patched RVOTs; median follow-up 360 days (IQR, 164–525)	Procedural success 53/53 (100%); prestenting performed in 45 patients	Median peak instantaneous RVOT gradient decreased from 23.5 mmHg (IQR, 10–53) to 10 mmHg (IQR, 5–16); moderate pulmonary regurgitation in three patients during follow-up	Multicenter observational cohort with heterogeneous substrates, limited follow-up, and no comparative or independently adjudicated analysis
Pulmonary; mixed RVOT substrates	SAPIEN 3/Ultra; [6]	840 treated; median follow-up 20.3 months, with estimates to 6 years	Successful implantation in 827/840 (98.5%); serious periprocedural adverse events 3.9%, including death in 0.6%	Median post-procedural gradient 6 mmHg (IQR 2–12); absent/trivial PR in 96.4%	Six-year cumulative incidence: endocarditis 3.8% (95% CI, 0.0–8.4%), pulmonary valve replacement 8.0% (95% CI, 1.2–14.8%), and thrombosis 0.7% (95% CI, 0.0–1.3%)
Pulmonary; RV–PA conduits or surgical bioprostheses	Melody; [56]	171 enrolled and 150 implanted; median follow-up 8.4 years	Enrollment-to-implantation proportion should not be interpreted as procedural success because of trial selection	Median discharge mean RVOT gradient 17 mmHg (IQR 12–22)	At 10 years, freedom from death 90%, reoperation 79%, any reintervention 60%, TPV dysfunction 53%, and TPV-related endocarditis 81%

Abbreviations: CI, confidence interval; IQR, interquartile range; LVOTO, left ventricular outflow tract obstruction; MAC, mitral annular calcification; MR, mitral regurgitation; NYHA, New York Heart Association; PR, pulmonary regurgitation; PVL, paravalvular leak; RV–PA, right ventricle-to-pulmonary artery; RVOT, right ventricular outflow tract; THV, transcatheter heart valve; TPV, transcatheter pulmonary valve; TR, tricuspid regurgitation.

## Data Availability

No new data were created or analyzed in this study. Data sharing is not applicable to this article.

## Supplementary Materials

The following supporting information can be downloaded at: https://www.mdpi.com/article/10.3390/bioengineering13090957/s1, Figure S1: Volume of published Myval experience according to non-aortic position; Figure S2: Study-level changes in transvalvular gradients after Myval implantation.

## Abbreviations

The following abbreviations are used in this manuscript:

Abbreviation	Definition
3D	Three-dimensional
AML	Anterior mitral leaflet
BATMAN	Balloon-assisted translocation of the anterior mitral leaflet
CIED	Cardiac implantable electronic device
CMR	Cardiovascular magnetic resonance
CT	Computed tomography
DAPT	Dual antiplatelet therapy
ECG	Electrocardiography
HALT	Hypoattenuated leaflet thickening
HF	Heart failure
ID	Internal diameter
IQR	Interquartile range
LAD	Left anterior descending coronary artery
LAMPOON	Laceration of the anterior mitral leaflet to prevent outflow obstruction
LV	Left ventricle or left ventricular
LVOT	Left ventricular outflow tract
LVOTO	Left ventricular outflow tract obstruction
MAC	Mitral annular calcification
MP35N	Nickel–cobalt–chromium–molybdenum alloy
MR	Mitral regurgitation
MVARC	Mitral Valve Academic Research Consortium
MViR	Mitral valve-in-ring
MViV	Mitral valve-in-valve
neo-LVOT	Predicted residual left ventricular outflow tract after transcatheter mitral valve replacement
NYHA	New York Heart Association
OAC	Oral anticoagulation
PA	Pulmonary artery
PCI	Percutaneous coronary intervention
PET	Polyethylene terephthalate
PR	Pulmonary regurgitation
PVL	Paravalvular leak
QRS	QRS complex
RA	Right atrium or right atrial
RCA	Right coronary artery
RV	Right ventricle or right ventricular
RV–PA	Right ventricle-to-pulmonary artery
RVOT	Right ventricular outflow tract
SAPT	Single antiplatelet therapy
TEE	Transoesophageal echocardiography
THV	Transcatheter heart valve
TMVI	Transcatheter mitral valve implantation
TMVR	Transcatheter mitral valve replacement
TPVI	Transcatheter pulmonary valve implantation
TR	Tricuspid regurgitation
TTE	Transthoracic echocardiography
TVARC	Tricuspid Valve Academic Research Consortium
TViR	Tricuspid valve-in-ring
TViV	Tricuspid valve-in-valve
VARC-3	Valve Academic Research Consortium 3
ViMAC	Valve-in-mitral annular calcification
VKA	Vitamin K antagonist
