# Supplementary material for: Myval Beyond the Aortic Valve: Device–Anatomy Interaction, Procedural Strategy, and Clinical Evidence in Mitral, Tricuspid, and Pulmonary Positions
Source: Bioengineering (Basel). 2026 Aug 22;13(9):957. doi: 10.3390/bioengineering13090957 (PMC13603376; doi:10.3390/bioengineering13090957)
Supplement: Supplementary file 1 [file bioengineering-13-00957-s001.zip › bioengineering-4492975-supplementary.pdf]

Supplementary Figures

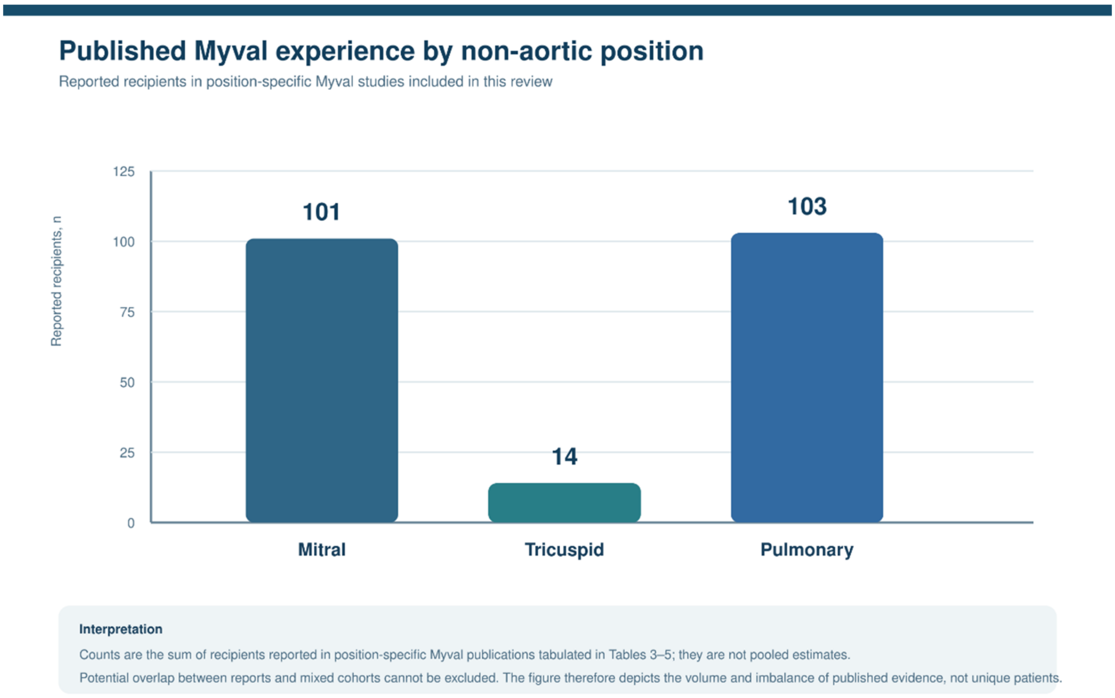

Supplementary Figure S1. Volume of published Myval experience according to non-aortic position. Bars show the sum of recipients reported in position-specific Myval publications included in Tables 3–5: mitral, 101; tricuspid, 14; and pulmonary, 103. Counts are descriptive and are not pooled estimates or unique-patient denominators, because overlap between reports and mixed cohorts cannot be excluded. The figure illustrates the marked scarcity of published tricuspid evidence.

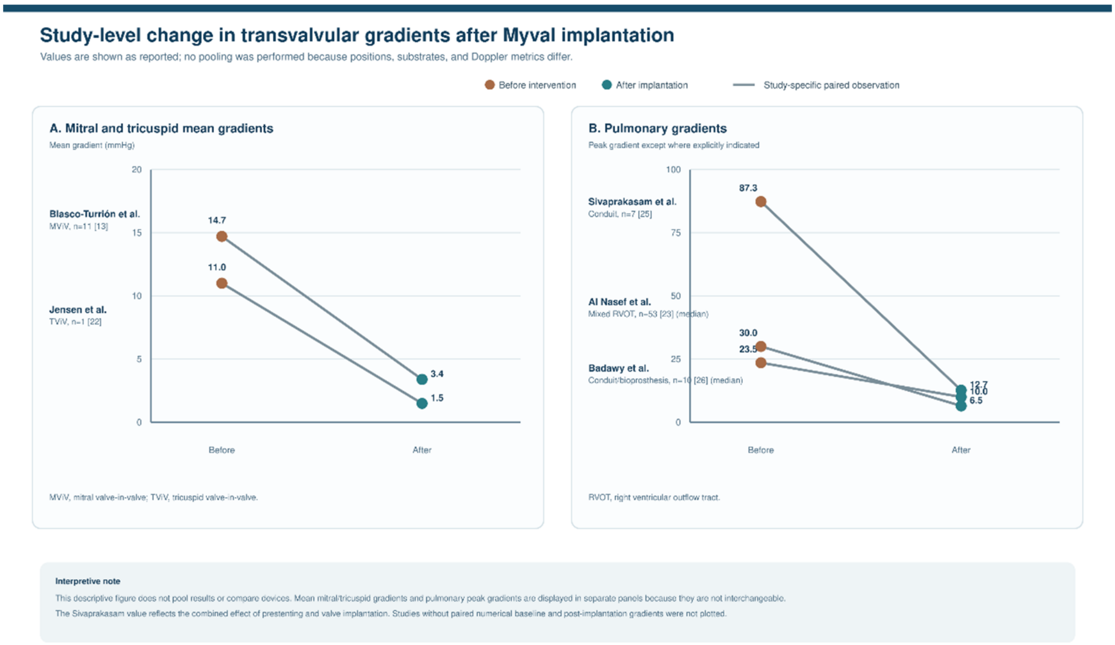

Supplementary Figure S2. Study-level changes in transvalvular gradients after Myval implantation. (A) Mean mitral and tricuspid gradients in studies providing paired numerical values [13,22]. (B) Pulmonary gradients reported using study-specific Doppler metrics: mean conduit gradient in Sivaprakasam et al. and peak instantaneous RVOT or conduit gradients in Al Nasef et al. and Badawy et al. [23,25,26]. Plotted values are study-level point estimates; measures of dispersion and assessment timing are provided in Tables 3–5. No pooling or cross-study statistical comparison was undertaken because valve position, substrate, timing, and Doppler metric differed. The Sivaprakasam values reflect the combined effects of prestenting and valve implantation.
